# Tissue-specific knockout in the *Drosophila* neuromuscular system reveals ESCRT’s role in formation of synapse-derived extracellular vesicles

**DOI:** 10.1371/journal.pgen.1011438

**Published:** 2024-10-10

**Authors:** Xinchen Chen, Sarah Perry, Ziwei Fan, Bei Wang, Elizabeth Loxterkamp, Shuran Wang, Jiayi Hu, Dion Dickman, Chun Han

**Affiliations:** 1 Weill Institute for Cell and Molecular Biology and Department of Molecular Biology and Genetics, Cornell University, Ithaca, New York, United States of America; 2 Department of Neurobiology, University of Southern California, Los Angeles, California, United States of America; National University of Singapore, SINGAPORE

## Abstract

Tissue-specific gene knockout by CRISPR/Cas9 is a powerful approach for characterizing gene functions during development. However, this approach has not been successfully applied to most *Drosophila* tissues, including the *Drosophila* neuromuscular junction (NMJ). To expand tissue-specific CRISPR to this powerful model system, here we present a CRISPR-mediated tissue-restricted mutagenesis (CRISPR-TRiM) toolkit for knocking out genes in motoneurons, muscles, and glial cells. We validated the efficacy of CRISPR-TRiM by knocking out multiple genes in each tissue, demonstrated its orthogonal use with the Gal4/UAS binary expression system, and showed simultaneous knockout of multiple redundant genes. We used CRISPR-TRiM to discover an essential role for SNARE components in NMJ maintenance. Furthermore, we demonstrate that the canonical ESCRT pathway suppresses NMJ bouton growth by downregulating retrograde Gbb signaling. Lastly, we found that axon termini of motoneurons rely on ESCRT-mediated intra-axonal membrane trafficking to release extracellular vesicles at the NMJ. Thus, we have successfully developed an NMJ CRISPR mutagenesis approach which we used to reveal genes important for NMJ structural plasticity.

## Author summary

We have developed a tissue-specific Cas9 toolkit that enables gene knockout specifically in motor neurons, glial cells, and muscle cells, the three principle cell types of the *Drosophila* peripheral motor system. Complementary to existing RNAi methods, this versatile tissue-specific knockout system offers unique advantages for dissecting gene functions at the neuromuscular junction (NMJ). Using these tools, we discovered that SNARE-mediated secretory pathways are required to maintain the integrity of the NMJ and that ESCRT components play critical yet differential roles in the biogenesis of extracellular vesicles, bouton growth, and membrane turnover at the NMJ. This CRISPR toolkit can be applied to study many biological questions in the neuromuscular system.

## Introduction

Characterization of developmental mechanisms often involves loss-of-function (LOF) analysis of genes in organisms. Besides the more traditional methods of LOF, such as whole-organismal mutations and RNA interference (RNAi), tissue-specific mutagenesis through the CRISPR/Cas9 system has recently emerged as another powerful approach [1–5]. In this approach, either Cas9 or gRNAs (or both) are expressed in a tissue-specific manner, so that CRISPR-mediated mutagenesis of the gene of interest (GOI) occurs only in the desired tissues [1,2,6,7]. Although tissue-specific CRISPR can be very effective for studying gene function, its successful application has been limited to a small number of *Drosophila* tissues, including the germline [1,8], sensory neurons [9–12], prothoracic gland [13], mushroom body neurons [14], imaginal tissues [7,9,10], epidermal cells [9,10], circadian neurons [15,16] and cardiomyocytes [17]. The *Drosophila* neuromuscular junction (NMJ) is a powerful model for studying many biological processes, such as axon development, synaptogenesis, synaptic function and plasticity, and locomotion [18–20]. Although CRISPR has been used to tag endogenous proteins at the NMJ [21], tissue-specific CRISPR has not yet been successfully applied to the fly NMJ.

NMJs are special synaptic connections formed between motor neurons and somatic muscles [22]. The axon termini of the motor neurons release extracellular vesicles (EVs) into the space between axons and muscles [23–26]. Outside the nervous system, EVs can originate from many types of cells and can carry diverse cargos made of all four types of biomolecules (carbohydrates, lipids, proteins, and nucleic acids) [27]. These vesicles may function in long-distance signal transduction and cell-cell communication and have been shown to play important roles in many physiological and pathological processes [28,29]. EVs at *Drosophila* NMJs contain several protein and nucleic acid cargos [23,30] and regulate synaptic structure and activity by mediating axon-to-muscle signal transduction [23,25]. So far, studies of the *Drosophila* NMJ have revealed the requirement of several genes related to intracellular vesicle trafficking and the endocytic pathway in the biogenesis of EVs [24,26,29]. However, the mechanisms of EV biogenesis at the fly NMJ are not fully understood, and many pathways that are important for EV production in mammalian cells have not been examined in *Drosophila*. One example is the endosomal sorting complex required for transport (ESCRT) pathway [31], which comprises five distinct complexes (ESCRTs -0, -I, -II, and -III, and Vps4) that sequentially sort monoubiquitinated membrane cargo and mediate membrane fission in late endosomes and at the plasma membrane.

In this study, we generated a tissue-specific CRISPR toolkit for knocking out genes in motor neurons, glia, and somatic muscles, the three cell types of the *Drosophila* NMJ. This toolkit is based on CRISPR-mediated tissue-restricted mutagenesis (CRISPR-TRiM), a method using tissue-specific expression of Cas9 and ubiquitously expressed gRNAs to knock out genes in the desired tissue [9]. We validated the efficacy of gene knock-out (KO) in each of the three tissues. Using these tools, we examined the roles of the Soluble N-ethylmaleimide-sensitive factor attachment protein receptor (SNARE) pathway and the ESCRT machinery in NMJ morphogenesis. Our results reveal a requirement for the SNARE pathway in NMJ maintenance and critical functions of ESCRT in EV biogenesis, axonal growth, and intra-axonal membrane trafficking.

## Results

### Tissue-specific Cas9 lines for motor neurons, glia, and muscle cells

To apply CRISPR-TRiM in tissues relevant to NMJ biology, we generated several Cas9 lines that are expressed in motoneurons, glial cells, or somatic muscles (S1A Fig) by Gal4-to-Cas9 conversion [10], enhancer-fusion [9], or CRISPR-mediated knock-in (KI). For motoneurons, we generated *wor-Cas9*, which should be active in neuronal progenitor cells [32], and three lines (*OK371-Cas9*, *OK6-Cas9*, and *OK319-Cas9*) that should be expressed in post-mitotic motoneurons [10,33–36]. For glial cells, we previously made *repo-Cas9* [10]; for this study, we also generated *gcm-Cas9* by inserting Cas9 into the *glial cells missing* (*gcm)* locus through CRISPR-mediated KI (S1A and S1B Fig). *gcm-Cas9* is predicted to be active in glial progenitor cells [37,38]. Lastly, we made a muscle-specific *mef2-Cas9* by converting *mef2-Gal4* [10] (S1A Fig).

**Fig 1:**
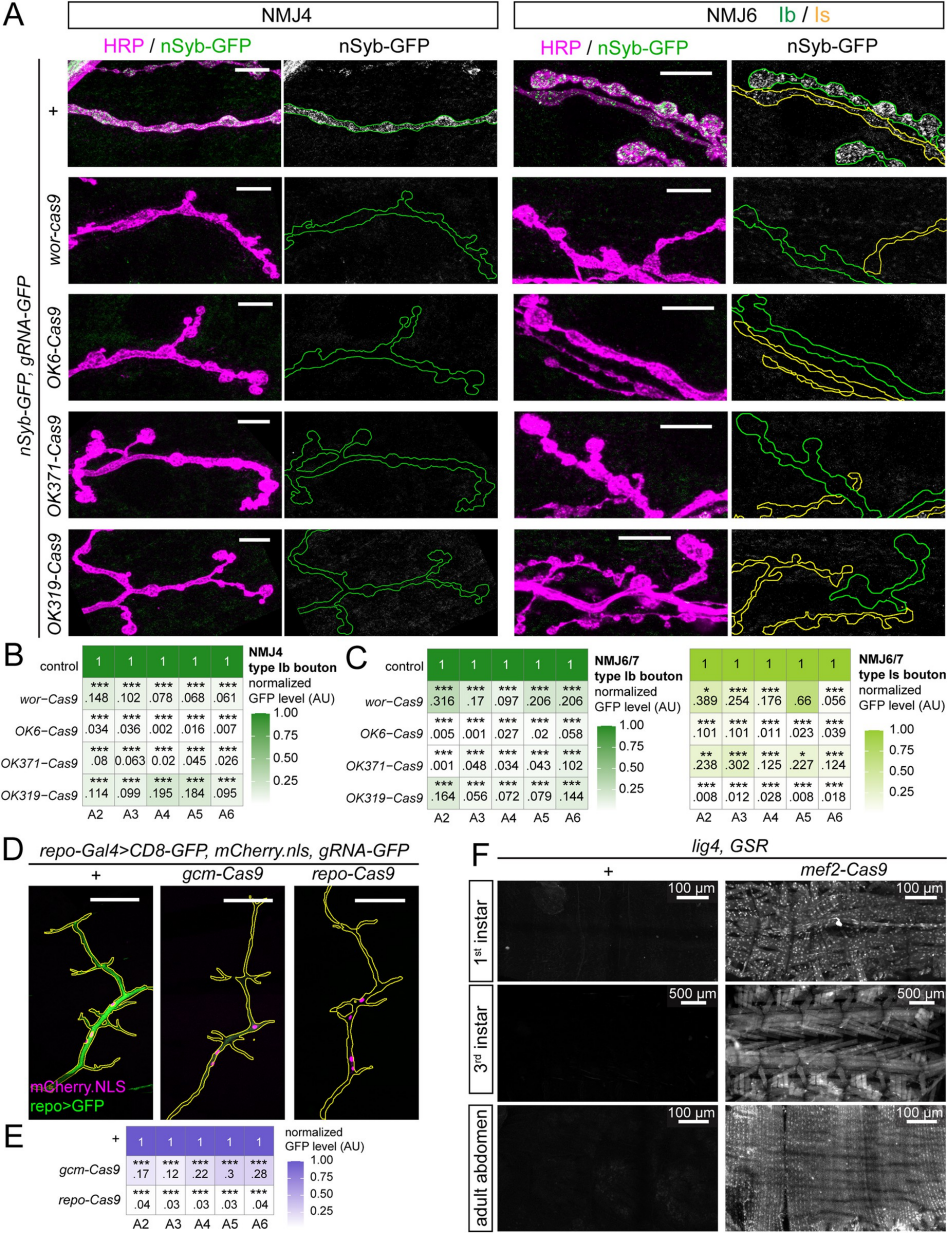
Tissue-specific Cas9 activity patterns characterized by Cas9 reporters. (A) Activity patterns of neuron-specific Cas9 lines at the NMJs of muscle 4 (NMJ4) and muscle 6/7 (NMJ6/7) as visualized by the negative tester *nSyb-GFP; gRNA-GFP*. The control cross does not have a Cas9. Type Ib boutons of NMJ4 and NMJ6/7 and Type Is boutons of NMJ6/7 were examined. Scale bar: 10 μm. (B–C) Quantification of presynaptic GFP intensity at NMJ4 (B) and NMJ6/7 (C). ***p≤0.001; one-way ANOVA. *p* values were adjusted by Bonferroni post hoc method. See S2 Table for sample sizes. (D) Activity patterns of glia-specific Cas9 lines in intersegmental nerves (ISNs) characterized with a negative tester *repo-Gal4*, *UAS-CD8-GFP*, *UAS-mCherry*.*nls*, *gRNA-GFP*. Scale bar: 10 μm. (E) Quantification of glial GFP intensity along ISNs. ***p≤0.001; one-way ANOVA. *p* values were adjusted by Bonferroni post hoc method. See S2 Table for sample sizes. (F) The activity pattern of muscle-specific *Mef2-Cas9*, characterized by a single strand annealing (SSA)-based positive tester *GSR*. The non-homologous end joining (NHEJ)-deficient *lig4* mutation was combined with *GSR* to increase the frequency of SSA and thus the reliability of GSR labeling. Upper panel, 1^st^ instar larva; middle panel, 3^rd^ instar larva fillet; lower panel, adult abdomen.

To evaluate the mutagenesis efficiency in motoneurons, we generated a Cas9 negative tester *nSyb-tdGFP; gRNA-GFP*. *nSyb-tdGFP* is expressed in all neurons [9] and labels motoneuron axon terminals (Fig 1A and 1B). *gRNA-GFP* expresses two gRNAs ubiquitously, targeting GFP and EGFP coding sequences [10]. Activities of different Cas9 transgenes can be compared by measuring remaining GFP fluorescence in relevant cells when tested with such negative testers [9]. We examined Ib boutons innervating muscles 4 and 6, as well as Is boutons on muscle 6, in A2-A6 segments. As expected, we observed strong reduction of GFP signals across segments at these NMJs with all four motoneuron or progenitor-cell Cas9 lines (Figs 1A–1C and S1C). However, we also noticed some differences among the Cas9 lines: *OK371-Cas9* and *OK6-Cas9* resulted in more consistent GFP reduction in Ib boutons than *wor-Cas9* and *OK319-Cas9*, but in Is boutons, *OK319-Cas9* and *OK371-Cas9* reduced GFP more consistently.

To test glial Cas9 lines, we generated a second negative tester, *gRNA-GFP UAS-mCherry*.*NLS; repo-Gal4 UAS-CD8-GFP*. This tester additionally expresses nuclear mCherry in glia, which is important for locating glia when GFP is not visible. We measured GFP levels along intersegmental nerve tracts in segments A2-A6 (Fig 1D). While *repo-Cas9* resulted in consistently strong (≥96%) reduction of GFP, *gcm-Cas9* caused weaker (70–88%) and more variable reduction (Figs 1E and S1E).

To visualize *mef2-Cas9* activity, we used GFP SSA reporter (GSR) [10], which ubiquitously expresses a transgene containing an interrupted EGFP-2A-nGFP coding sequence. Cas9 activity is reported by GFP reconstitution, which occurs only when Cas9 causes a breakage at the site of interruption and triggers DNA repair by single-strand annealing (SSA) [10]. With GSR, we detected robust GFP signals in somatic muscles from newly hatched to wandering 3^rd^ instar larvae and in the adult abdomen (Fig 1F).

While using GSR to examine motoneuron Cas9 drivers, we observed *OK6-Cas9* activity in many peripheral tissues, including epidermal cells and trachea (S1D Fig). Using the lineage-tracing Gal4 reporter *tubP(FRT*.*stop)Gal4 UAS-Flp UAS-mCD8*::*GFP* [10], we confirmed that *OK6-Gal4* is also active in these peripheral tissues during development (S1D Fig), which is consistent with a previous report [39].

Thus, we generated several Cas9 lines that are active in motor neurons, peripheral glia, or somatic muscles and thus are appropriate for tissue-specific mutagenesis at the NMJ.

### Efficient tissue-specific KO of genes at the larval NMJ

**Fig 2:**
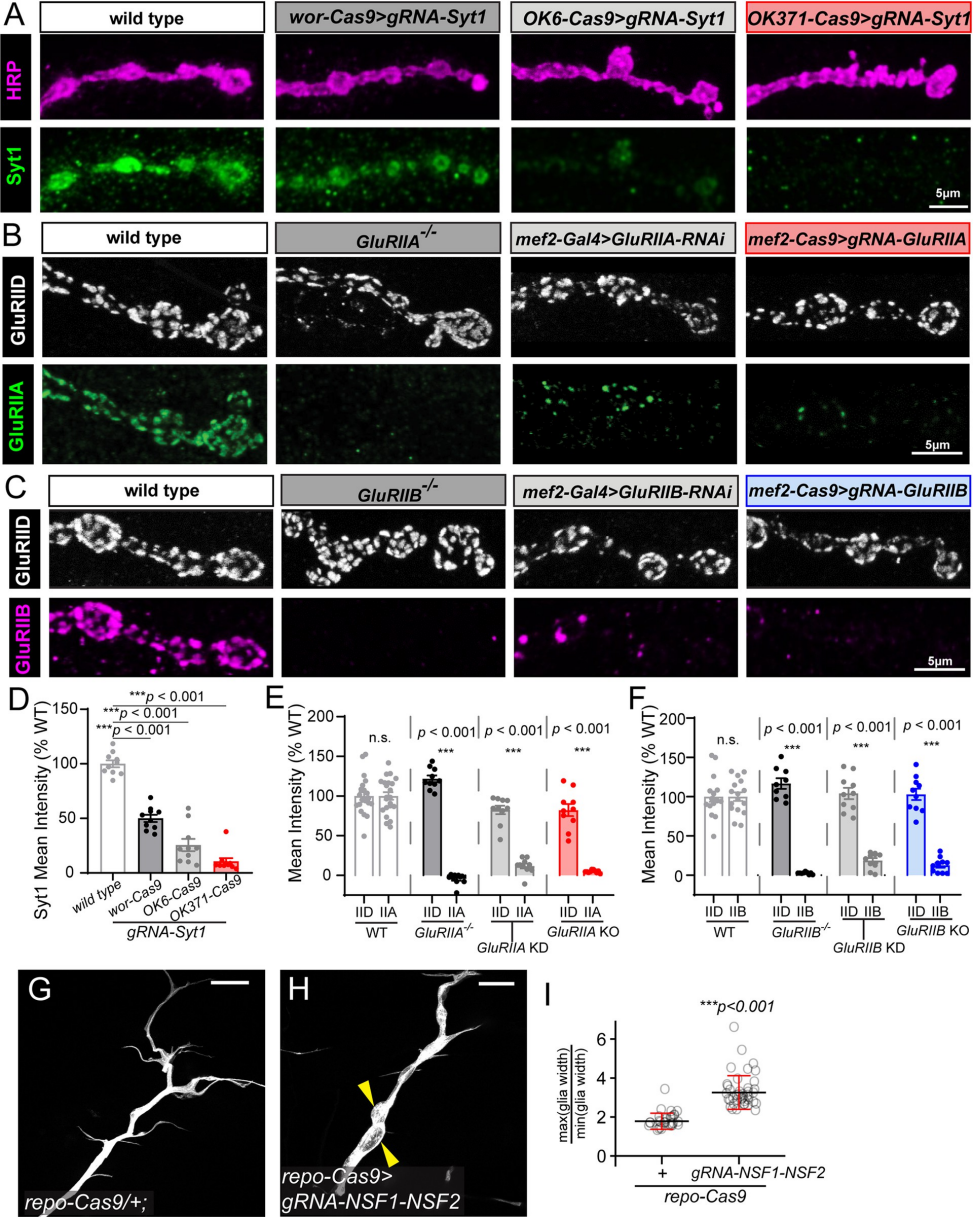
Efficient gene knockout induced by CRISPR-TRiM in the larval neuromuscular system. (A) *Syt1* knockout (KO) in motoneurons by *wor-Cas9*, *OK6-Cas9* and *OK371-Cas9*. The Syt1 protein is detected by antibody staining. The axon membrane is visualized by HRP staining. (B) Comparison of different methods to remove GluRIIA expression in muscles: whole animal *GluRIIA* mutant (2^nd^ panel), muscle-specific RNAi (3^rd^ panel), and muscle-specific CRISPR KO (4^th^ panel). The GluRIIA protein is detected by antibody staining. GluRIID staining serves as an internal control. (C) Comparison of similar methods as (B) to induce *GluRIIB* loss-of-function in muscles. GluRIIB protein level is detected by antibody staining. GluRIID level is unaffected and serves as an internal control. (D–F) Mean intensity of staining of Syt1 (D), GluRIIA and GluRIID (E), and GluRIIB and GluRIID (F) in the indicated genotypes. One-way ANOVA, p < 0.0001 for all 3 datasets compared to wild type; WT, n = 10; *wor-Cas9>gRNA-Syt1*, n = 10; *OK6-Cas9>gRNA-Syt1*, n = 10; *OK371-Cas9>gRNA-Syt1*, n = 10; WT, n = 22; *GluRIIA*^*-/-*^, n = 10; *Mef2-Gal4>GluRIIA-RNAi*, n = 10; *Mef2-Cas9>gRNA-GluRIIA*, n = 10; WT, n = 16; *GluRIIB*^*-/-*^, n = 9; *Mef2-Gal4>GluRIIB-RNAi*, n = 10; *Mef2-Cas9>gRNA-GluRIIB*, n = 10. All NMJs shown are Ib boutons at NMJ4 in A2-A4 segments. No data were thrown out in the analysis. (G–H) Intersegmental nerve glia in the control (G) and glial-specific KO of *NSF1/NSF2* by *repo-Cas9* (H). Glial cells are labeled with *repo-Gal4>UAS-CD4-tdTomato*. Yellow arrowheads indicate glial enlargement. Scale bar: 50 μm. (I) The ratio of maximal/minimal nerve thickness in control and glial *NSF1/NSF2* KO. The segment before the first major branch in each intersegmental nerve (ISN) is examined (see S2D Fig). ***p≤0.001; t-test. *repo-Cas9*: n = 29; *repo-Cas9>gRNA-NSF1-NSF2*: n = 41.

To evaluate the efficiency of gene KO in the motor system using CRISPR-TRiM, we generated or obtained ubiquitously expressed gRNAs for several well-studied genes and crossed them to appropriate Cas9 lines. We compared the efficiency of *wor-Cas9*, *OK6-Cas9*, and *OK371-Cas9* in knocking out *Synaptotagmin 1* (*Syt1*) (Fig 2A), which encodes a transmembrane Ca^2+^ sensor for synchronous synaptic vesicle fusion [40]. Crossing to *wor-Cas9* resulted in 49.5% reduction of Syt1 protein at muscle 4 Ib boutons as assayed by immunostaining (Fig 2A and 2D), while *OK6-Cas9* and *OK371-Cas9* caused 74.5% and 89.5% Syt1 reduction, respectively (Fig 2A and 2D). To assess physiological consequences of CRISPR-mediated Syt1 knockout, we performed evoked recordings of *e*xcitatory postsynaptic potentials (EPSPs) from muscles 6 and 7. EPSP amplitudes were reduced to 53% and 47% of the wildtype (WT) level in *OK6-Cas9-* and *OK371-Cas9*-mediated KO, respectively, as expected, whereas *wor-Cas9* did not result in significant EPSP reduction (S2A Fig), perhaps due to the more variable KO efficiency of *wor-Cas9* at muscle 6 (S1C Fig). Consistent with the function of Syt1, miniature excitatory postsynaptic potentials (mEPSP) in these animals were unaltered (S2B Fig).

The efficiency of muscle-specific *mef2-Cas9* was evaluated using gRNAs for two *Drosophila* muscle-specific glutamate receptor subunits, GluRIIA and GluRIIB. In both cases, *mef2-Cas9* efficiently eliminated expression of these proteins in muscle fibers (Fig 2B and 2C). CRISPR-TRiM generated comparable or stronger reduction than RNAi-induced knockdown (KD) as assayed by immunostaining (Fig 2E and 2F). However, while electrophysiological recordings showed trends of reduced mEPSP amplitude in *GluRIIA* KO and enhanced mEPSP amplitude in *GluRIIB* KO (S2C Fig), the changes were not significant, suggesting that the residual GluR protein was still sufficient to mediate persistent functionality.

To determine the efficiency of *repo-Cas9*, we used previously published gRNAs targeting both *comatose* (*comt or Nsf1*) and *N-ethylmaleimide-sensitive factor 2* (*Nsf2*) [9], which encode two redundant NSF proteins involved in disassembly of SNARE complexes after vesicle fusion [41–43]. When paired with a neuronal Cas9, this gRNA transgene results in strong dendrite reduction of *Drosophila* somatosensory neurons [9,10]. As vesicle fusion is an essential “house-keeping” function, we anticipated that loss of both *Nsf* genes in glia should also disrupt glial morphology and function. Indeed, larvae containing both *repo-Cas9* and *gRNA-Nsf1-Nsf2* showed locomotion defects and died at the late third instar stage. In these animals, the glia wrapping motoneuron axons formed enlarged compartments that were absent in *repo-Cas9* controls (Fig 2G and 2H) resulting in a greater ratio between the maximal and minimal widths of the glial tract (Figs 2I and S2D). These results suggest that *repo-Cas9* can efficiently induce biallelic mutations of two genes simultaneously in glia.

Altogether, the above results demonstrate that Cas9 expressed by motoneurons, muscles, and glial cells can result in efficient tissue-specific gene KO for studying motoneuron development and NMJ biology.

### CRISPR-TRiM reveals a requirement for SNARE components in NMJ maintenance

To investigate NMJ morphogenesis with CRISPR-TRiM, we next examined the role of the secretory pathway mediated by SNARE proteins. Because many SNARE genes play essential housekeeping functions in all cells, we reason that their LOF in motoneurons should result in morphological defects at the NMJ. In addition, because these genes are expected to be expressed early in neuronal lineages and are prone to perdurance, testing them with CRISPR-TRiM may reveal differences in the timing of action among the Cas9 drivers in motoneurons. Snap25, Snap24, and Snap29 are *Drosophila* SNARE proteins in the Qbc subgroup that mediate fusion of secretory vesicles with the plasma membrane [44]. We have previously generated a transgene expressing six multiplexed gRNAs targeting all three *Snap* genes simultaneously and have showed that it efficiently suppressed dendrite growth of *Drosophila* sensory neurons with an appropriate Cas9 [9]. As the three proteins play partially redundant functions, LOF of the entire gene group is required to reveal morphological defects in neurons [9]. Thus, *Snap* genes are a good test case for simultaneous gene KO by CRISPR-TRiM at the NMJ.

**Fig 3:**
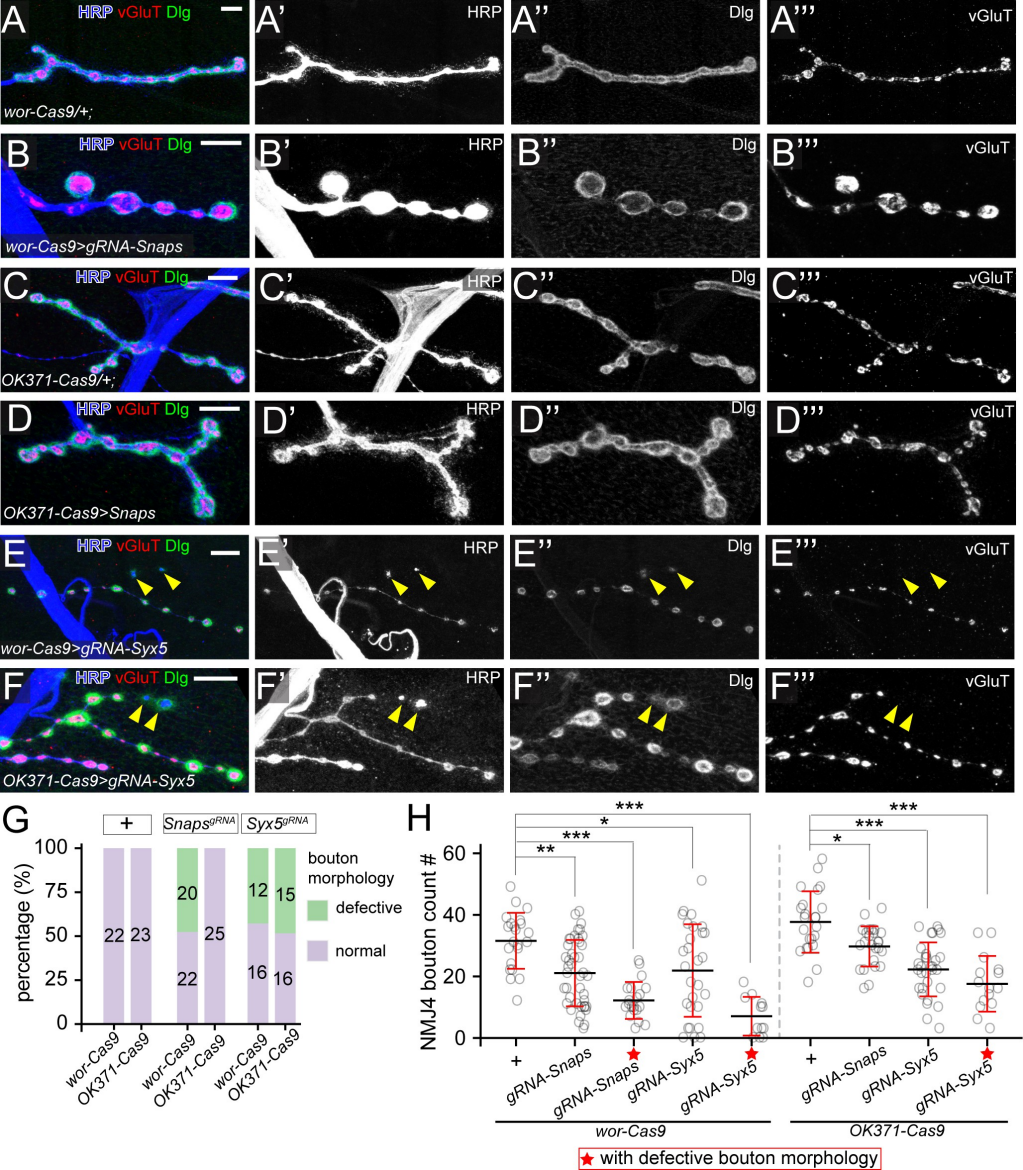
SNARE components are required for NMJ maintenance. (A–C) Boutons of *wor-Cas9* (A), triple KO of *Snap24*/*Snap25*/*Snap29* by *wor-Cas9* (B) and *Syx5* KO by *wor-Cas9* (C). Scale bar: 10μm. (D–F) Boutons of *OK371-Cas9* (D), triple KO of *Snap24*/*Snap25*/*Snap29* by *OK371-Cas9* (E), and *Syx5* KO by *OK371-Cas9* (F). In (A–F), neuronal membrane is labeled by HRP staining, presynaptic density is marked by vesicular glutamate transporter (vGluT) antibody staining and subsynaptic reticulum (SSR) is labeled by Disc Large (Dlg) antibody staining. Scale bar: 10μm. (G) Penetrance of observable bouton morphology defects in 6 genotypes shown in (A–F). Numbers indicate the sample size of each genotype. (H) Bouton numbers of genotypes shown in (A–F). ***p≤0.001; **p≤0.01; *p≤0.05; One-way ANOVA. Each circle represents an NMJ: *wor-Cas9*, n = 22; *Snaps*^*wor-Cas9*^, n = 42; ★*Snaps*^*wor-Cas9*^, n = 20; *Syx5*^*wor-Cas9*^, n = 28; ★*Syx5*^*wor-Cas9*^, n = 12; *OK371-Cas9*, n = 23; *Snaps*^*OK371-Cas9*^, n = 25; *Syx5*^*OK371-Cas9*^, n = 31; ★*Syx5*^*OK371-Cas9*^, n = 15, *p* values are from multiple comparison test using Bonferroni adjustment. All boutons were from NMJ4 in segments A2-A4. Groups with red stars contain only NMJs with observable bouton defects.

Combining this multiplexed Snap24-Snap25-Snap29 (Snaps) gRNA with *wor-Cas9* resulted in apparent locomotion defects since early larval stage, and the larvae died between late 3rd instar and wandering 3^rd^ instar stages. At 96 h after egg laying (AEL), 48% of NMJs innervating muscle 4 in these larvae showed morphological defects characterized by large and round boutons with thin connections (Fig 3A, 3B and 3G), suggesting that simultaneous KO of all three genes occurred in a mosaic pattern. On average, we observed 33% reduction of the bouton number at all NMJs examined, but the reduction is increased to 61% when only those morphologically defective NMJs were considered (Fig 3H). In addition, large vesicles with strong HRP staining were found in each bouton (S3A and S3B Fig), suggesting accumulation of intra-axonal membranes. In contrast, when *OK371-Cas9* was used to knock out *Snap* genes, we observed only a moderate reduction of the bouton number across all NMJs (Fig 3C, 3D, 3G and 3H). The difference between *wor-Cas9* and *OK371-Cas9* is consistent with our previous results that a Cas9 expressed in neuronal precursor cells is necessary for revealing strong LOF phenotypes of *Snap* genes in somatosensory neurons [9].

*Syx5* encodes a Q-SNARE protein that mediates ER to Golgi transport, an important step in the secretory pathway [45]. Using CRISPR-TRiM, we have previously shown that *Syx5* KO in sensory neurons leads to severe dendrite reduction [10]. Combining the same *gRNA-Syx5* with either *wor-Cas9* or *OK371-Cas9* resulted in severe morphological defects at 43% and 48% muscle 4 NMJs (Fig 3E–3H), suggesting uneven KO. The morphological defects include thinned axons, smaller bouton size, reduced bouton number, and in severe cases, detachment of boutons from axons. The detached boutons showed no vGlut staining and weaker-than-normal Dlg staining (Fig 3E and 3F), indicating NMJ degeneration [46]. Although the NMJs showed a wide range of bouton reduction, the ones with obvious morphological aberrations displayed severer and much more consistent reductions (Fig 3H). In comparison, *Syx5* KD by *OK371-Gal4* only showed moderate bouton reduction and no other morphological defects (S3C–S3F Fig).

Together, the above data suggest that SNARE-mediated secretory pathways are required for structural establishment or maintenance of NMJs and that CRISPR-TRiM can reveal LOF phenotype of essential and redundant genes at the NMJ.

### CRISPR-TRiM reveals roles of ESCRT in motoneuron morphogenesis and EV biogenesis

**Fig 4:**
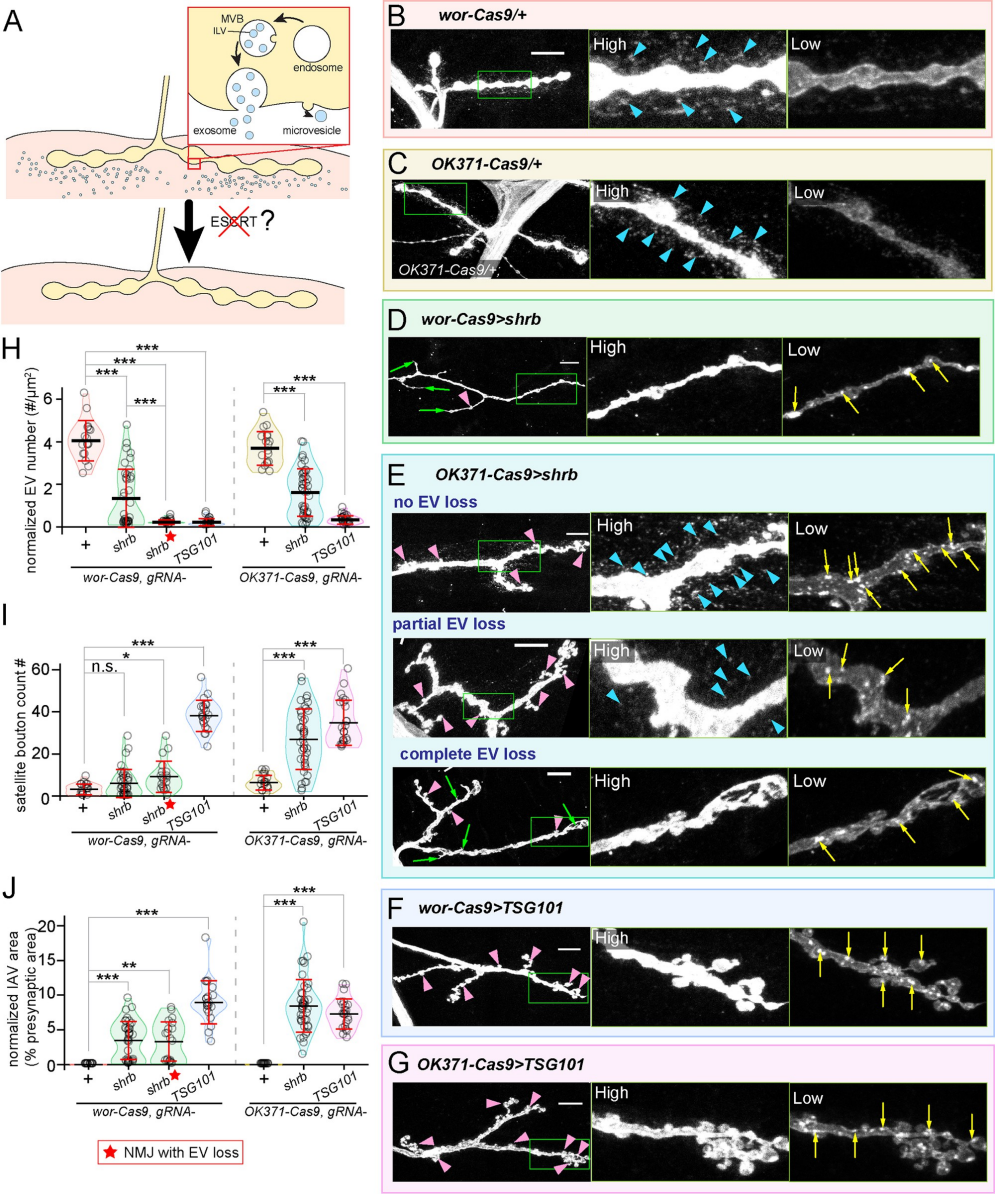
CRISPR-TRiM reveals roles of ESCRT in motoneuron morphogenesis and EV biogenesis. (A) A diagram of possible routes of EV biogenesis at the NMJ and the experimental design. (B–G) NMJs in *wor-Cas9* (B) and *OK371-Cas9* (C) controls, *shrb* KO by *wor-Cas9* (D) and *OK371-Cas9* (E), and *TSG101* KO by *wor-Cas9* (F) and *OK371-Cas9* (G). Motoneurons are visualized by HRP staining. “High” and “Low” panels show the zoomed-in views of the area enclosed by the green box imaged with high (to visualize EVs) and low (to visualize IAVs) intensity settings. Three images of *OK371-Cas9>shrb* in (E) show different degrees of EV loss. Blue arrowheads in (B), (C) and (E) indicate the EVs surrounding the presynaptic compartment. Yellow arrows in (D), (E), (F) and (G) indicate IAVs. Green arrows in (D) and (E) indicate filamentous protrusions formed by the presynaptic membrane. Pink arrowheads in (D–G) indicate satellite boutons. Scale bar: 10μm. (H-J) EV numbers (H, normalized by the presynaptic area), satellite bouton numbers (I), and IAV areas (J, normalized by the presynaptic area) from various genotypes. ***p≤0.001, **p≤0.01, *p≤0.05, n.s., not significant. One-way ANOVA. Each circle represents an NMJ: *wor-Cas9*, n = 17; *shrb*^*wor-Cas9*^, n = 36; ★*shrb*^*wor-Cas9*^, n = 19; *TSG101*^*wor-Cas9*^, n = 21; *OK371-Cas9*, n = 18; *shrb*^*OK371-Cas9*^, n = 41; *TSG101*^*OK371-Cas9*^, n = 23; *p* values are from multiple comparison test using Bonferroni adjustment. All boutons were from NMJ4 in segments A2-A4. Groups with red stars contain only NMJs with strong EV loss.

*Drosophila* motoneurons release extracellular vesicles into the synaptic cleft at NMJs. Studies in mammalian cells have uncovered important roles of ESCRT complexes in EV biogenesis [47–49], but whether the ESCRT pathway is involved in EV release at the *Drosophila* NMJ remains unknown. Thus, we investigated the roles of Shrub (Shrb), Tumor susceptibility gene 101 (TSG101) and ALG-2 interacting protein X (ALiX), three components at different steps of the ESCRT pathway, in EV biogenesis at the NMJ using CRISRT-TRiM (Fig 4A).

Shrb is the *Drosophila* homolog of Snf7, a central subunit of the ESCRT-III complex, which is responsible for outward budding and fission of vesicles from late endosomes and the plasma membrane [31,50]. We generated *gRNA-shrb* and knocked out *shrb* using *wor-Cas9* and *OK371-Cas9* (Fig 4A). In the WT, membranes originated from the axon can be detected by HRP staining [51,52], including EVs that appear as numerous puncta surrounding axon termini of motoneurons (Fig 4B and 4C, blue arrowheads). *wor-Cas9* together with *gRNA-shrb* resulted in several morphological defects at Ib boutons of the muscle 4 NMJ, including reduction of EVs (Fig 4D and 4H) and appearance of bright HRP-positive puncta inside distal axons (3.43% presynaptic area), which are obvious under a lower detection setting (yellow arrows in Fig 4D and 4J). Notably, 53% of all NMJs showed complete or near complete loss of EVs (Fig 4H, asterisk), while the rest showed mild or no EV loss (Fig 4H), suggesting that severe *shrb* LOF occurred in only about half NMJs. The NMJs exhibiting extreme EV loss also grew filamentous branches (Fig 4D, green arrows) and showed a mild (2.92-fold) increase of satellite boutons (pink arrowhead in Fig 4D and 4I), even though the increase of satellite boutons was not statistically significant when all NMJs were considered. We refer to the bright HRP-positive puncta as intra-axonal vesicles (IAVs), which were absent in control neurons (Fig 4B and 4J). Neuroglian (Nrg), a known EV cargo at *Drosophila* larval NMJ [53], was accumulated at these IAVs (S4A Fig), indicating mis-trafficking of EV-destined cargos.

When *shrb* was knocked out by *OK371-Cas9*, we also observed EV reduction, satellite bouton increase, and IAV accumulation at muscle 4 NMJs (Fig 4E and 4H–4J). However, the severity of each phenotype shows a different distribution from that induced by *wor-Cas9*. EV levels at these NMJs were distributed more evenly from complete loss to almost wildtype levels (Fig 4E and 4H). In contrast, the increases of satellite bouton number (4.25-fold) and IAV accumulation (8.45% presynaptic area) were much more pronounced with *OK371-Cas9* than with *wor-Cas9* (Fig 4E, 4I and 4J). As a comparison, we also examined *shrb* KD using *OK371-Gal4*, which resulted in comparable increases in satellite bouton number (3.83-fold) and IAV accumulation (6.86% presynaptic area) to *OK371-Cas9 gRNA-shrb* but a more complete and consistent EV loss (78.7% reduction) (S4B–S4E Fig). We interpret the extreme phenotypes (complete EV loss, moderate increases of satellite bouton and IAVs, and prominent filamentous protrusions) in *wor-Cas9 gRNA-shrb* as strong *shrb* LOF defects and the phenotypes in *OK371-Cas9 gRNA-shrb* (moderate EV loss and strong increases of satellite bouton and IAVs) as moderate defects. *shrb* KD appears to cause intermediate defects between the two. Thus, *shrb* KD resulted in less variable phenotypes than KO, but pre- and post-mitotic KO revealed a broader spectrum of LOF phenotypes.

We next examined TSG101, an ESCRT-I complex component that functions in endosomal cargo sorting and exosome biogenesis [54–56]. Unlike *gRNA-shrb*, which caused variable NMJ defects from NMJ to NMJ, *gRNA-TSG101* induced complete penetrance with both *wor-Cas9* and *OK371-Cas9* (Fig 4F–4J). *wor-Cas9 gRNA-TSG101* showed a 95% reduction of EVs, a 12.2-fold increase in satellite bouton number and IAV accumulation (8.96% area) (Fig 4F and 4H–4J) as compared to the control. *TSG101* KO by *OK371-Cas9* also showed near-complete EV loss (91% reduction), satellite bouton increase (5.5 folds), and IAV accumulation (7.29% area) (Fig 4G and 4H–4J). Thus, for both Cas9s, *TSG101* KO produced consistent, strong, and comparable phenotypes, suggesting that both pre-mitotic and post-mitotic KO of *TSG101* may reveal the null phenotype. As a comparison, *TSG101* KD by *OK371-Gal4* resulted in weaker EV reduction (80%), satellite bouton increase (3.5 folds), and IAV accumulation (5.96% area) (S4B–S4E Fig).

Lastly, we knocked out *ALiX*, which encodes a BRO1 domain-containing protein that can recruit the ESCRT-III complex, as ALiX has been shown to be involved in EV biogenesis in mammalian cells [49,57,58]. ALiX functions upstream of ESCRT-III in an alternative pathway to the canonical pathway mediated by ESCRT-0, I, and II complexes [57–59]. We generated *gRNA-ALiX* and validated its efficiency using the Cas9-LEThAL assay (See Methods) [9]. However, even pre-mitotic *ALiX* KO by *wor-Cas9* did not exhibit any noticeable morphological defects (S4F–S4H Fig), suggesting that motor neurons use the canonical ESCRT pathway, not the ALiX-assisted pathway, to generate EVs.

Together, the above data suggest that the canonical ESCRT pathway is required for both EV biogenesis and for suppressing the growth of satellite boutons. However, the severities of these two phenotypes do not always correlate, suggesting that they may be controlled by divergent pathways downstream of TSG101 and Shrb.

### ESCRT KO induces satellite bouton overgrowth through aberrant BMP signaling

**Fig 5:**
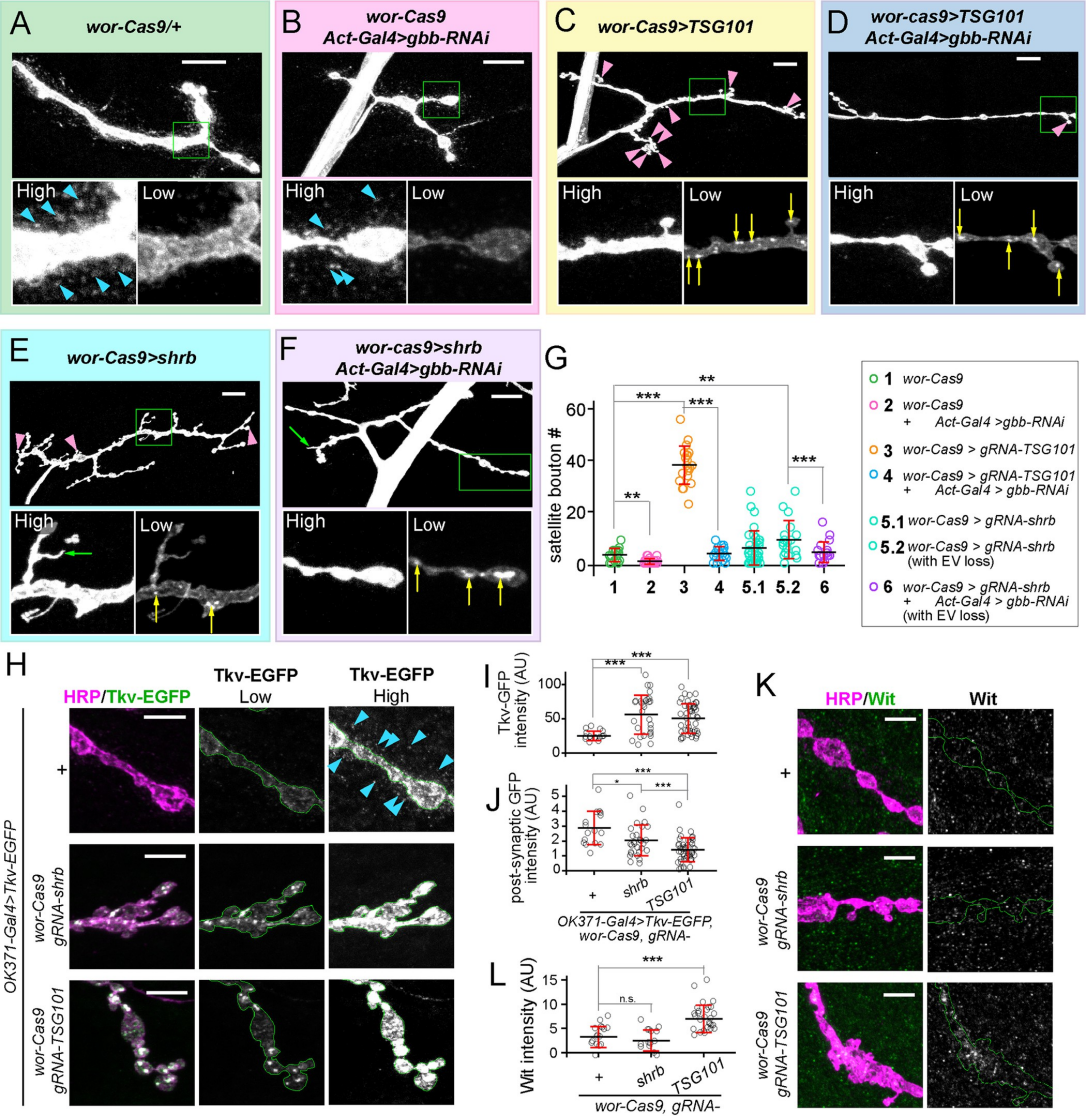
ESCRT LOF causes satellite bouton overgrowth by regulating BMP signaling. (A–F) NMJ morphologies in the control (A), global *gbb* KD (B), *TSG101* KO by *wor-Cas9* (C), motoneuronal *TSG101* KO combined with global *gbb* KD (D), *shrb* KO by *wor-Cas9* (E) and motoneuronal *shrb* KO combined with global *gbb* KD (F). Motoneurons are visualized by HRP staining. “High” and “Low” panels show the zoomed-in views of the area enclosed by the green box. The same NMJ is imaged with both high and low intensity settings. Pink arrowheads indicate satellite boutons. Yellow arrows indicate IAVs. Blue arrowheads indicate EVs. Green arrowheads indicate protrusions from axons. Scale bar: 10μm. (G) satellite bouton numbers in the indicated genotypes. ***p≤0.001, **p≤0.01, One-way ANOVA. Each circle represents an NMJ: *wor-Cas9*, n = 17; *gbb-RNAi*^*Act-Gal4*^, n = 25; *TSG101*^*wor-Cas9*^, n = 21; *TSG101*^*wor-Cas9*^ */ gbb-RNAi*^*Act-Gal4*^, n = 20; *shrb*^*wor-Cas9*^, n = 36; *shrb*^*wor-Cas9*^ with EV loss, n = 19; *shrb*^*wor-Cas9*^ */ gbb-RNAi*^*Act-Gal4*^ with EV loss, n = 20; between-group *p* values are from multiple comparison test using Bonferroni adjustment. The datasets of *wor-Cas9>gRNA-shrb* and *wor-Cas9>gRNA-TSG101* are the same as in Fig 4. (H) Tkv-EGFP signals in control (upper panel), *shrb* KO (middle panel) and *TSG101* KO (lower panel) neurons. Blue arrowheads indicate EVs. Scale bar: 10μm. (I) Mean Tkv-EGFP intensity inside boutons in control, *shrb* KO and *TSG101* KO animals. ***p≤0.001, One-way ANOVA. Each circle represents an NMJ: *Gal4*^*OK371*^*>Tkv-EGFP*, n = 16; *Gal4*^*OK371*^*>Tkv-EGFP*, *Cas9*^*wor*^*>shrb*^*gRNA*^, n = 29; *Gal4*^*OK371*^*>Tkv-EGFP*, *Cas9*^*wor*^*>TSG101*^*gRNA*^, n = 46, between-group *p* values are from multiple comparison test using Bonferroni adjustment. (J) Mean postsynaptic Tkv-EGFP intensity in control, *shrb* KO and *TSG101* KO animals. ***p≤0.001, *p≤0.05, One-way ANOVA. Each circle represents an NMJ: *Gal4*^*OK371*^*>Tkv-EGFP*, n = 16; *Gal4*^*OK371*^*>Tkv-EGFP*, *Cas9*^*wor*^*>shrb*^*gRNA*^, n = 29; *Gal4*^*OK371*^*>Tkv-EGFP*, *Cas9*^*wor*^*>TSG101*^*gRNA*^, n = 46, between-group *p* values are from multiple comparison test using Bonferroni adjustment. (K) Wit staining in control (upper panel), *shrb* KO (middle panel) and *TSG101* KO (lower panel) neurons. Scale bar: 5 μm. (L) Mean Wit level inside boutons in control, *shrb* KO and *TSG101* KO animals. ***p≤0.001, One-way ANOVA. Each circle represents an NMJ: *wor-Cas9*, n = 16; *Cas9*^*wor*^*>shrb*^*gRNA*^, n = 14; *Cas9*^*wor*^*>TSG101*^*gRNA*^, n = 28, between-group *p* values are from multiple comparison test using Bonferroni adjustment.

Bouton growth at the *Drosophila* NMJ relies on both anterograde wingless signaling and retrograde BMP signaling [60–64]. In the retrograde BMP signaling pathway, the muscle-derived BMP ligand Glass-bottom boat (Gbb) binds to presynaptic type II BMP receptors Wishful thinking (Wit). Wit then binds and phosphorylates type I BMP receptor Thick veins (Tkv)/Saxophone (Sax) to form an activated signaling complex [63,65]. The BMP ligand-receptor complex stabilizes boutons locally via LIM1 kinase [66] and are also endocytosed and transported to the neuronal soma where it phosphorylates mothers against decapentaplegic (Mad) to promote bouton growth through transcriptional regulation [60,63,65,67]. Defective endocytic pathway in neurons result in excessive satellite boutons due to persistent ligand-receptor interaction [67]. Because of the role of the ESCRT in sorting both loaded and empty receptors into the multiple vesicular bodies (MVBs) for later degradation [54,68–70], loss of ESCRT may impair degradation of Gbb receptors, leading to persistent Gbb signaling. To test this possibility, we asked whether the satellite bouton increase associated with ESCRT LOF depends on Gbb by combining neuronal KO of ESCRT (via *wor-Cas9*) with global KD of *gbb*. Consistent with the role of Gbb in stimulating bouton growth [60], global *gbb* KD by *Act-Gal4* (Fig 5B) caused 28.6% reduction of total bouton number (S5A Fig) as compared to the control. Strikingly, *gbb* KD reduced satellite boutons in *TSG101* KO to the control level (Fig 5C, 5D and 5G). Because satellite bouton increase in *wor-Cas9 gRNA-shrb* was observed only at the NMJs that also showed EV loss (Figs 4I and 5G), we counted the satellite boutons of the NMJs devoid of EVs in *shrb* KO with additional *gbb* KD. The satellite boutons were also reduced to the control level (Fig 5E–5G). These data suggest that Gbb signaling is indeed responsible for the increase of satellite boutons in ESCRT LOF. The filamentous protrusions associated with *shrb* KO were still present after *gbb* KD (Fig 5F), suggesting that these structures are independent of Gbb signaling. To determine the source of Gbb that is responsible for the satellite boutons increases in ESCRT KO, we next knocked down *gbb* in motoneurons (by *OK371-Gal4*) and muscles (by *mef2-Gal4*) separately. Neuronal- and muscle-specific *gbb* KD caused 19.1% and 53.3% reduction of the satellite boutons, respectively, in *wor-Cas9 gRNA-TSG101* (S5B–S5C Fig), suggesting that both neuronal and muscle Gbb contributes to the bouton overgrowth.

The above results led us to hypothesize that the signaling mediated by Gbb receptors is potentiated upon the loss of ESCRT. To directly examine Gbb receptors and the downstream signaling, we first expressed Tkv-EGFP in motoneurons, which by itself did not cause bouton overgrowth (S5D Fig). Tkv-EGFP was found to localize on the bouton membrane (Fig 5H), as previously reported [71]. Interestingly, we also found Tkv-EGFP in EVs at WT NMJs (S5E Fig), suggesting that Tkv is also an EV cargo. Similar to Nrg (S4A Fig), Tkv-EGFP was accumulated in IAVs in both *shrb* and *TSG101* KO (via *wor-Cas9*), leading to 2.3-fold and 2.1-fold increases of overall EGFP intensity in boutons, respectively (Fig 5H and 5I). Additionally, ESCRT KO resulted in reductions of post-synaptic Tkv-EGFP, mirroring those of EVs (Fig 5H and 5J). We next examined Wit levels using immunostaining. Despite the low signals of Wit staining, we were able to detect a mild (2.1-fold) increase of Wit in boutons of *TSG101* KO (Fig 5K and 5L). To assay Gbb signaling, we examined phosphorylated Mad (pMad) in the larval ventral nerve cord (VNC), where motoneuron somas reside, and in NMJ boutons by immunostaining. Consistent with elevated Gbb signaling, we observed mild increases of nuclear pMad in VNCs of *shrb* and *TSG101* KO (S5F and S5G Fig) and in boutons of *TSG101* KO (S5H and S5I Fig). Together, these data support that ESCRT LOF potentiates Gbb signaling through aberrant receptor degradation or disposal.

Lastly, to understand whether the roles of TSG101 and Shrb in EV biogenesis and intra-axonal membrane turnover are related to Gbb signaling, we also examined EV and IAV levels in *TSG101* and *shrb* KO combined with *gbb* KD. Global *gbb* KD did not affect the EV level by itself (Figs 5B and S5A), nor did it rescue the EV loss in *TSG101* or *shrb* KO (Figs 5D, 5F and S5A), confirming that EV biogenesis and satellite bouton growth are controlled by separate pathways downstream of ESCRT. Interestingly, *gbb* KD caused a 40.0% reduction of IAVs in *TSG101* KO but did not significantly change IAV levels in *shrb* KO (S5A Fig). These results suggest that IAV accumulation in *TSG101* KO is partially due to Gbb signaling.

## Discussion

### CRISPR-TRiM is a versatile tool for dissecting gene function in the NMJ system

Tissue-specific mutagenesis by CRISPR is a powerful approach for dissecting gene functions in animal development [1,4,9–12,14–16,72]. This approach directly disrupts the coding sequence of, and thus knocks out, the gene of interest in specific somatic cells. However, its successful application in the *Drosophila* NMJ system has not been demonstrated previously. In this study, we developed a Cas9 collection for applying CRISPR-TRiM in motor neurons, somatic muscles, and glia cells, the three principal cell types that make up the NMJ. Using these tools, we demonstrate the effectiveness of gene KO in each tissue and reveal the role of the SNARE pathway in NMJ maintenance and the roles of the ESCRT pathway in NMJ morphogenesis.

Compared to LOF by RNAi, the CRISPR-TRiM method offers two major advantages. First, it is relatively easy to express multiple gRNAs to knock out several genes simultaneously, and thus this method is particularly useful for studying redundant genes, as exemplified by the analyses of *Nsf* and *Snap* genes. In comparison, almost all existing RNAi transgenes target single genes, and it is either labor intensive or impossible to combine them because they are inserted in few common loci. Second, because mutagenesis occurs individually in each cell and could yield different degrees of LOF, CRISPR-TRiM has the potential to reveal both weak and strong LOF phenotypes of the gene of interest (GOI) using a single gRNA transgene. This property can be useful for dissecting complex functions of genes, as demonstrated by *OK371-Cas9 gRNA-shrb*. Unlike Gal4-dependent CRISPR strategies [1,7], the tissue-specific Cas9s in our CRISPR-TRiM method can be used orthogonally with binary expression systems, as exemplified by simultaneous *TSG101* KO in neurons and tissue-specific KD of *gbb*. Thus, the tools we reported here enable a versatile CRISPR toolkit to analyze gene function in the NMJ system.

### Factors determining the efficacy of CRISPR-TRiM

To reveal the null phenotype of a gene at the single cell level, biallelic LOF mutations need to be generated early in the cell’s lineage [9]. In practice, multiple factors can influence the timing and the nature of mutations, and thus the efficacy of tissue-specific KO.

First, the expression timing, duration, and strength of the Cas9 can largely affect the extent of LOF. Cas9s that are expressed in neural stem cells or progenitor cells can result in earlier mutations than those that are expressed in postmitotic neurons. An example is that, with the same *gRNA-shrb*, *wor-Cas9* caused more severe EV loss than *OK371-Cas9*. On the other hand, post-mitotic Cas9s presumably have stronger and more long-lasting expression than precursor-cell Cas9s. This property could be important for using gRNAs that require higher Cas9 activity or take longer to generate DNA breaks. *gRNA-GFP* and *gRNA-Syt1* may belong to this category.

Second, the efficiency of gRNAs critically affects the outcome of KO. The gRNA efficiency is affected by multiple factors, including the target sequence [73,74], the gRNA construct design [6,7,75,76], the accessibility of the target sequence [74,77–79], and the functional significance of the mutated amino acids. To increase the success rate of gRNAs, we recommend the following guidelines: (1) Select target sequences predicted to have high efficiency scores by multiple experimentally validated algorithms [73,79–82]; (2) Choose two gRNAs for each gene of interest, targeting the earlier part of the coding sequence to increase the possibility of early truncations; (3) Avoid target sequences that are subject to frequent single nucleotide polymorphisms, which can render gRNAs ineffective; (4) Select Cas9 cut sites near sequences that encode conserved amino acid residues to increase the chance of LOF mutations; (5) Construct gRNAs in high-efficiency expression vectors such as pAC-U63-gRNA2.1 [10]; (6) Validate the DNA cutting efficiency of transgenic gRNAs using the Cas9-LEThAL assay [9]. Nevertheless, high mutagenic efficiency of gRNAs does not always translate into a high penetrance of LOF phenotypes. If in-frame mutations at the target site do not completely disrupt a protein’s function, a portion of the cells carrying even biallelic mutations will display no or hypomorphic phenotypes. This scenario could have contributed to the motor neurons that showed no defects in *shrb* and *Syx5* KO and possibly the variable phenotypes of *OK371-Cas9 gRNA-shrb* neurons. However, variable phenotypes for the same gene can be advantageous for revealing multiple facets of a gene’s function, as in this case.

Lastly, the expression timing and product stability of the GOI can affect the severity of the LOF phenotype. For genes that are expected to express late in the cell lineage, such as only in differentiating neurons, post-mitotic Cas9 can be early enough for causing LOF [9]. *Syt1* may fall in this category. In contrast, house-keeping genes are typically expressed earlier than tissue-specific Cas9s and are thus more prone to perdurance effects [9]. For these genes, early expressing Cas9s should be more effective than late Cas9s, as in the case of the KO for *shrb*, *Syx5*, and *Snap* genes. However, if the mRNA/protein products of the GOI are rapidly turned over, even house-keeping genes could be effectively removed by a late expressing Cas9. This scenario may explain *TSG101* KO results.

In summary, the efficacy of CRISPR-TRiM is influenced by Cas9 expression pattern, gRNA efficiency, and the characteristics of the GOI. In conducting CRISPR-TRiM, optimal results can be achieved by choosing the appropriate combinations of Cas9 and gRNAs. Interpretation of the results should also take consideration of the property of the GOI.

### The ESCRT pathway controls multiple aspects of NMJ morphogenesis

The ESCRT complex is known to play diverse roles in different cellular contexts, including sorting of endosomal proteins [50,83–87], biogenesis of MVBs [88–91], biogenesis of exosome [27] and extracellular vesicles [56,92], membrane repair [93–96], autophagy [97–101] and cytokinesis [102,103]. In neurons, ESCRT is involved in neurite growth control [104,105], synapse maintenance [106], neurite and synaptic pruning [107–109], and neurotransmission regulation [110]. In this study, by examining *shrb* and *TSG101* KO, we uncovered several aspects of NMJ morphogenesis controlled by the ESCRT pathway, namely EV biogenesis, satellite bouton growth, and intra-axonal membrane trafficking. Specifically, disruptions of the ESCRT pathway resulted in EV loss, overgrowth of satellite boutons, and accumulation of IAVs. Our results suggest that these phenotypes are controlled by both shared and separate pathways downstream of ESCRT components.

First, the ESCRT pathway suppresses satellite bouton growth by downregulating Gbb signaling. Because ESCRT plays a general role in sorting signaling receptors to ILVs of MVBs for subsequent delivery to lysosomes [88], in the absence of ESCRT, ligand-bound BMP receptors may be more stable and thus can sustain the signaling longer, as has been shown for TGF-β receptors [68]. In addition, like other receptors [54,69,70], in the absence of ESCRT, unbound BMP receptors may accumulate in the cell and are recycled back to the axon membrane, sensitizing motor neurons to Gbb. Either effect could lead to potentiation of Gbb signaling and satellite bouton overgrowth.

Second, we found that the ESCRT pathway is essential for EV biogenesis at the NMJ. However, our data suggest that EV loss and satellite bouton overgrowth are two uncorrelated defects: The NMJs that showed complete or near complete loss of EVs in *shrb* KO still display the full spectrum of satellite bouton phenotypes (Fig 4H and 4I). In addition, global *gbb* KD in ESCRT LOF completely suppressed satellite bouton formation but had no impact on EV release, further confirming that the EV loss and satellite bouton overgrowth are controlled by two separate pathways. The importance of ESCRT in EV biogenesis has been reported in other systems previously [27,48,49,56]. EVs are generated through either fusion of MVBs with the plasma membrane (exosomes) or outward budding of vesicles from the plasma membrane (microvesicles) [27], two processes that both require the ESCRT machinery [111]. However, the roles of ESCRT in the biogenesis of axon-derived EVs have not been characterized previously. Our results confirmed that the EVs at the NMJ also depend on the ESCRT.

Third, we found that ESCRT prevents accumulation of IAVs by both Gbb-dependent and independent mechanisms. ESCRT is known to be important for endomembrane turnover by generating ILVs that are subsequently degraded in the lysosome [112]. For this reason, disruptions of ESCRT could cause accumulation of late endosomes and give rise to IAVs. The enrichment of the anti-HRP epitope suggests that these vesicles may normally feed into degradative compartments. Interestingly, reducing Gbb levels in *TSG101* KO animals largely alleviated this phenotype. It is possible that Gbb signaling increases IAV formation by stimulating biogenesis and delivery of membranes to axons termini and further clogging the system.

Lastly, our data provide interesting clues about how EVs are generated by axons. We found that the EV cargo Nrg is accumulated at IAVs in *shrb* KO, suggesting that the EV cargo is sorted to these endosomal compartments before release. Thus, it appears that at least some EVs are exosomes generated through the MVB pathway. However, this observation does not rule out the possibility of EV biogenesis through microvesicle budding at the plasma membrane.

### Shared and separate pathways downstream of ESCRT components at the NMJ

Our analyses of three ESCRT components show that their LOF does not produce identical phenotypes. First, while mutant neurons of *shrb* and *TSG101* both show near complete EV loss, *shrb* mutant neurons with extreme morphological defects did not exhibit as strong increases of satellite boutons and IAV accumulation as *TSG101* mutant neurons. Instead, they grew filamentous membrane protrusions, which were absent in *TSG101* mutant neurons. In addition, whereas *TSG101* KO causes both Gbb-dependent and Gbb-independent IAV accumulation, the milder IAV accumulation in *shrb* KO is largely Gbb-independent. Thus, the Gbb signaling seems to contribute to the NMJ defects of *TSG101* KO much more strongly than to those of *shrb* KO. These differences could be due to TSG101 and Shrb functioning at different steps of signaling receptor processing on endosomal membranes. Even though the Gbb receptor Tkv accumulates at the boutons of both *shrb* and *TSG101* KOs, it may exist in different states so that less Tkv protein in *shrb* KO is signaling capable. Alternatively, ESCRT-III is required for more molecular processes than those involving ESCRT-I [31,113–115], so that the additional defects caused by *shrb* LOF counteract the effects of receptor accumulation and dampen the overall membrane accumulation at the axon terminal. Second, we found that *ALiX* KO does not affect NMJ morphology. ALiX acts in parallel with ESCRT-I to direct ubiquitinated cargo to ESCRT-III [57]. Like ESCRT-I, ALiX has also been shown to be involved in EV biogenesis [57,58]. However, we found that ALiX does not contribute to EV biogenesis at the NMJ, suggesting that ALiX’s role in EV formation may be context-dependent.

## Materials and methods

### *Drosophila* stocks and culture

The details of fly strains used in this study are listed in S1 Table. All crosses were set up in standard yeast-sugar fly food and kept at 25°C and 60% humidity, with 12 h light/dark cycle until examination.

### Molecular cloning

*gcm-Cas9*: Two gRNA spacer sequences targeting the 5’UTR immediately before the start codon of *gcm* were first cloned into pAC-U63-tgRNA-nlsBFPnls (Addgene 169029) [116] according to published protocols. The resulting plasmid was digested by PstI and assembled with three DNA fragments through NEBuilder HiFi DNA assembly to make a *gcm* gRNA-donor vector. The three DNA fragments are a 5’ homology arm (827 bp immediately before *gcm* start codon) in which the gRNA target sequences were mutated, a Cas9-T2A fragment PCR amplified from pDEST-APIC-Cas9 (Addgene 121657) [9], and a 3’ homology arm (966 bp immediately after the start codon).

*gRNA transgenic vectors*: gRNA target sequences for genes of interest were selected using the CRISPOR server (http://crispor.gi.ucsc.edu/) and cloned into various gRNA vectors according to published protocols [9,10]. The gRNA target sequences are listed in Table 1, and the cloning vectors and the PCR templates are listed in Table 2.

**Table 1:** gRNA target sequences.

gene	target sequence 1	target sequence 2	target sequence 3
Syt1	GTATAATCTTCTTCTGTGTG	AGGAGGGTGACGAGGAGGAC	CGTGACGGTGATCCAAGCCG
GluRIIA	CAATCGCACCGACGTAATGT		
GluRIIB	GGTGTCTTCATTGGCGCCGC		
gcm	GTTAGTTTCAAGTTCACACG	TTACATAGACACATCAAAAA	
shrb	GACCACGATGAAGAATGCCG	GATCGGCGCCGAATGCCACT	
TSG101	GCAGGTCGTAGGTCAAGCTG	TACTTGGGCGAGGGTCTCCG	
ALiX	GATGGTCGCCCAAGCGCAGG	GAGTGGCCGGACATGCCTGG	

**Table 2:** gRNA expression vectors.

gRNA lnes	gRNA cloning vector	PCR template
gRNA-Syt1	pAC-U63-tgRNA-Rev	pMGC
gRNA-GluRIIA*	pCFD3	NA
gRNA-GluRIIB	pAC-U63-tgRNA-Rev	NA
gRNA-shrb	pAC-U63-tgRNA-Rev	pMGC
gRNA-TSG101	pAC-U63-tgRNA-Rev	pMGC
gRNA-ALiX	pAC-U63-tgRNA-Rev	pMGC

*gRNA-GluRIIA was generated by the TRiP CRISPR project and obtained from the Bloomington *Drosophila* Stock Center (BDSC).

Transgenic constructs were injected by Rainbow Transgenic Flies (Camarillo, CA, USA) to transform flies through φC31 integrase-mediated integration into attP docker sites.

### Generation of tissue-specific Cas9 lines

*wor-Cas9*, *OK6-Cas9*, and *mef2-Cas9* were converted from corresponding Gal4 lines using the HACK method as previously described [10]. GSR was used as the reporter for Cas9 conversion. A 2nd chromosomal donor was used to convert 2nd chromosomal Cas9 (*wor-Cas9* and *OK6-Cas9*) and a 3rd chromosomal donor was used to convert 3rd chromosomal Cas9 (*mef2-Cas9*).

To make *gcm-Cas9*, the *gcm* gRNA-donor vector was first inserted into the *VK3a* attP site [117] by φC31 integrase-mediated integration. This gRNA-donor transgene was then crossed to y[1] nos-Cas9.P[ZH-2A] w[*] [1] to induce homologous recombination in the germline of the progeny. The resulting male progeny were crossed to the Cas9 positive tester *Act-Gal4 UAS-EGFP; tub-Gal80 gRNA-Gal80* [9] for screening larvae that showed GFP signals in the brain. The larvae were recovered and used to derive isogenic *gcm-Cas9* strains by removing transgenic components on other chromosomes. 5 larvae showing identical GFP patterns in the brain were recovered from 172 larvae in total. The *gcm-Cas9* line is lethal as homozygotes.

### Validation of gRNA efficiency

The efficiency of transgenic gRNA lines was validated by the Cas9-LEThAL assay [9]. In this assay, homozygous males of the gRNA line are crossed to *Act-Cas9 w lig4* homozygous females. Because the *lig4* mutation is on the X chromosome, male progeny are hemizygous (*lig4/Y*) and thus is defective in NHEJ, while female progeny are heterozygous (*lig4/+*) and can repair DNA DSB. Efficient gRNAs for an essential gene should result in lethality of both males and females at a stage comparable to the lethal phase of homozygous null mutants of the same gene. Efficient gRNAs for non-essential genes should result in male lethality in late larval to pupal stages (due to the lack of DNA repair) and viable female adults (due to repair of DNA DSBs through NHEJ). *gRNA-Syt1*, *gRNA-GluRIIA*, *and gRNA-GluRIIB* crosses resulted in lethality before pupation; *gRNA-shrb* crosses resulted in lethality of all progeny in embryos; *gRNA-TSG101* crosses resulted in lethality of all progeny in 1^st^ to 2^nd^ instar larvae; *gRNA-ALiX* crosses resulted in lethality of male progeny from 3^rd^ instar larvae to pharate adults and viable and healthy female progeny. These results suggest that all gRNAs are efficient.

### Live imaging

Live imaging was performed as previously described [118]. Briefly, animals were reared at 25°C in density-controlled vials for 120 hours (wandering third instar). Larvae were mounted in glycerol and imaged using a Leica SP8 confocal microscope.

### Larval fillet preparation

Larval fillet dissection was performed on a petri dish half-filled with PMDS gel. Wandering third instar larvae were pinned on the dish in PBS dorsal-side up and then dissected to expand the body wall. PBS was then removed and 4% formaldehyde in PBS was added to fix larvae for 15 minutes at room temperature, or Bouin’s solution was added for 5 minutes at room temperature. Fillets were rinsed and then washed at room temperature in PBS for 20 minutes or until the yellow color from Bouin’s solution faded. After immunostaining, the head and tail of fillets were removed, and the remaining fillets were placed in SlowFade Diamond Antifade Mountant (Thermo Fisher Scientific) on a glass slide. A coverslip was lightly pressed on top. Larval fillets were imaged with 40× or 63× oil objectives using a Leica SP8 confocal microscope.

### Larval brain preparation

Larval brain dissection was performed as described previously [119]. Briefly, wandering 3rd instar larvae were dissected in a small petri dish filled with cold PBS. The anterior half of the larva was inverted, and the trachea and gut were removed. Samples were then transferred to 4% formaldehyde in PBS and fixed for 25 minutes at room temperature. Brain samples were washed with PBS. After immunostaining, the brains were placed in SlowFade Diamond Antifade Mountant on a glass slide. A coverslip was lightly pressed on top. Brains were imaged with both 20× and 40× oil objectives using a Leica SP8 confocal microscope.

### Immunohistochemistry

For larval brains: Following fixation, brains were rinsed and then washed twice at room temperature in PBS with 0.3% Triton-X100 (PBST) for 20 minutes each. Brains were then blocked in a solution of 5% normal donkey serum (NDS) in PBST for 1 hour. Brains were then incubated in the blocking solution with rat mAb 7E8A10 anti-elav (1:10 dilution, DSHB) or mouse mAb 8D12 anti-repo (1:20 dilution, DSHB) overnight at 4°C. Following incubation brains were then rinsed and washed in PBST 3 times for 20 minutes each. Brains were then incubated in a block solution containing a donkey anti-rat or donkey anti-mouse secondary antibody conjugated with Cy5 or Cy3 (1:400 dilution, Jackson ImmunoResearch) for 2 hours at room temperature. Brains were then rinsed and washed in PBST 3 times for 20 minutes each and stored at 4°C until mounting and imaging.

For larval fillets: following fixation, fillets were rinsed and then washed at room temperature in PBS. Fillets were then removed from PMDS gel and blocked in a solution of 5% normal donkey serum (NDS) in 0.2% PBST for 1 hour. Fillets were then incubated in the blocking solution with primary antibodies overnight at 4°C. Primary antibodies used in this study are mouse mAb BP 104 anti-Neuroglian (1:8 dilution, DSHB), mouse mAb 8B4D2 anti-GluRIIA (1:50, DSHB), rabbit anti-Syt (1:2500) [120], rabbit anti-GluRIIB (1:1000) [121], rabbit anti-vGluT (1:200 dilution, generated using the same peptide and approach described in [122]), guinea pig anti-GluRIID (1:1000) [120], mouse mAb 23C7 anti-wit (1:15 dilution, DSHB), and mouse mAb 4F3 anti-discs large (1:20 dilution, DSHB). Following incubation fillets were then rinsed and washed in PBST 3 times for 20 minutes each. Fillets were then incubated in a block solution containing fluorophore-conjugated conjugated secondary antibodies for 2 hours at room temperature. Secondary antibodies used in this study are: goat anti-HRP conjugated with Cy3 (1:200, Jackson ImmunoResearch), donkey anti-mouse secondary antibody conjugated with Cy5 or Alexa 488 (1:400, Jackson ImmunoResearch), donkey anti-rabbit secondary antibody conjugated with Cy5 or Alexa488 (1:400, Jackson ImmunoResearch), and rabbit polyclonal anti-GFP antibody conjugated with Alexa 488 (1:400, LifeTechnologies, A21311). Fillets were then rinsed and washed in PBST 3 times for 20 minutes each and stored at 4°C until mounting and imaging.

### Electrophysiology

Dissections were performed on wandering third instar larvae submerged in HL-3 saline, containing (in mM) 70 NaCl, 5 KCl, 10 MgCl_2_, 10 NaHCO_3_, 115 Sucrose, 5 Trehelose, 5 HEPES, and 0.4 CaCl_2_. Larvae were gently pinned down, cut down the midline, and the guts, trachea, and the CNS were removed, leaving the body wall and motor nerves. All recordings were performed on muscles 6 and 7 in hemisegments A2 and A3 of wandering third instar larvae in the same modified HL-3 saline. Spontaneous miniature excitatory post-synaptic potentials (mEPSPs) and evoked excitatory post-synaptic potentials (EPSPs) were obtained using an Olympus BX61 WI microscope with a water-40x/0.80 dipping objective, Axoclamp 900A amplifier, and Digidata 1440A. mEPSPs were recorded in the absence of stimulation, and average mEPSP amplitude per muscle (based on 1-minute gap-free recordings) was quantified using MiniAnalysis (Synaptosoft). EPSP data was acquired in sweeps of 20 stimulations at 0.5 Hz per muscle via an Iso-Flex stimulus isolator, which directly stimulated the motor neuron, and later analyzed using Clampfit (Molecular Devices). Further analysis for both mEPSP and EPSP data were performed in GraphPad 8 (Prism) and Excel (Microsoft) using one-way ANOVA. All recordings were monitored to ensure that the resting membrane potential was between -80 mV and -60 mV and muscle input resistance was between 5 MΩ and 35 MΩ.

### Image analysis and quantification

Images were analyzed on ImageJ/Fiji. Unless specified otherwise, muscle 4 NMJs of segments A2–A4 were imaged for quantification. The numbers of axial and satellite boutons were manually counted without blinding based on vGluT and Dlg staining. The thickness of intersegmental glia along the nerve tract was measured with the Local Thickness function, which gave rise to the maximal and minimal thickness for calculating the max/min ratio.

EV numbers and IAVs were quantified blindly in ImageJ using a batch script. Images were first segmented by the Trainable Weka Segmentation plugin based on HRP antibody staining. The machine learning-based program was first trained by several sample images to distinguish the background, presynaptic compartment, EVs, and IAVs. Then the models were applied to segment all the images in control and experimental groups. Single-pixel particles were removed from EV and IAV segmentations. A region of interest (ROI) was drawn to encircle the bouton to quantify in each image. The EV number and IAV coverage in the ROI were measured using Analyze Particles function in ImageJ. The EV number for each NMJ is normalized by the presynaptic membrane area. The IAV coverage (%) was generated by dividing the total area of IAVs by the corresponding presynaptic membrane area.

For measurement of GFP, Tkv-EGFP, wit and pMad levels at the NMJ, HRP staining was used to generate a mask of the bouton. Mean intensity of Tkv-EGFP, wit or pMad within the bouton and the ROI were quantified. For measuring pMad levels in the VNC, Elav staining was used to generate a mask of neuronal nuclei. Mean intensity of pMad in each nucleus was normalized to the intensity of Elav staining.

### Experimental design and statistical analysis

For all experiments, the control groups and the experimental groups were kept in the same growing conditions. The same dissection and staining procedures were applied to all the groups. The animals used for dissection were of the same age (~120h AEL wandering 3rd instar larva, unless specified otherwise). RStudio was used to perform one-way analysis of variance (ANOVA) and Student’s t-test where indicated. For experiments involving only two groups, a two-tailed t-test was used to compare the means. Non-equal variance was assumed. For experiments with more than two groups, one-way ANOVA was first applied to identify significantly different mean(s). After that, multiple comparisons were performed using the Bonferroni post hoc method.

## Supporting information

S1 Fig Cas9 activity patterns characterized by Cas9 reporters (related to Fig 1). (A) A diagram of the Cas9 lines made for this study and their targeting tissues. (B) A diagram of the generation of *gcm-Cas9* by CRISPR-mediated knock-in. A Cas9-2A coding sequence is inserted in-frame immediately after the start codon of *gcm*. TS1, target site 1. TS2, target site 2. HDR, homology-directed repair. (C) Scatter plots of all data shown in Fig 1A–1C. ***p≤0.001; one-way ANOVA. *p* values were adjusted by Bonferroni post hoc method. See S2 Table for sample sizes. (D) Comparison of *OK6-Gal4* and *OK6-Cas9* activity patterns in epidermal cells and trachea on the larval body wall. Activity pattern of *OK6-Cas9* is visualized by crossing to *lig4; GSR*. The non-homologous end joining (NHEJ)-deficient *lig4* mutation was combined with *GSR* to increase the frequency of SSA and thus the reliability of GSR labeling. *OK6-Gal4* activity pattern is visualized by *tubP(FRT*.*stop)Gal4*, *UAS-Flp*, *GFP*. Scale bar: 500 μm. (E) Scatter plots of all data shown in Fig 1D–1E. ***p≤0.001; one-way ANOVA. *p* values were adjusted by Bonferroni post hoc method. See S2 Table for sample sizes.
(TIF)

S2 Fig Gene knockout induced by CRISPR-TRiM in larval motoneurons leads to physiological defects (related to Fig 2). (A) Average evoked EPSP amplitudes comparing the motor neuron Cas9s *wor-Cas9/+;gRNA-Syt1*, *OK6-Cas9/+;gRNA-Syt1*, and *OK371-Cas9/+;gRNA-Syt1* to *w*^*1118*^ controls. ***p≤0.001; **p≤0.01; One-way ANOVA. Each dot represents an NMJ: *w*^*1118*^, n = 12; *wor-Cas9>gRNA-Syt1*, n = 14; *OK6-Cas9>gRNA-Syt1*, n = 9; *OK371-Cas9>gRNA-Syt1*, n = 9; *OK319-Cas9>gRNA-Syt1*, n = 13. (B) Average mEPSPs comparing lines listed in (A). No significant difference is found between the lines. One-way ANOVA. Sample sizes are the same as in (A). (C) Comparison of the muscle specific *Mef2-Cas9* to other methods of glutamate receptor loss-of-function in affecting mEPSPs. Electrophysiological recordings were conducted on muscles 6 and 7 in segments A2 and A3. *Mef2-Cas9/+;gRNA-GluRIIA/+* reduces mEPSP amplitude, as expected, though not significantly nor as robustly as *GluRIIA*^*pv3*^ null mutants or *Mef2-Gal4/+;UAS-GluRIIA-RNAi*. Similarly, *Mef2-Cas9/+;gRNA-GluRIIB/+* increases mEPSP amplitude to similar levels as *GluRIIB*^*sp5*^ null mutants and *Mef2-Gal4/+;UAS-GluRIIB-RNAi*, though again not statistically significantly different from control mEPSP amplitudes of *w*^*1118*^. ***p≤0.001, One-way ANOVA. Each dot represents an NMJ: *w1118*, n = 12; *GluRIIA*, n = 11; *Mef2-Cas9>gRNA-GluRIIA*, n = 12; *Mef2-Gal4>GluRIIA-RNAi*, n = 11; *GluRIIB*, n = 13; *Mef2-Cas9>gRNA-GluRIIB*, n = 14; *Mef2-Gal4>GluRIIB-RNAi*, n = 11. (D) The method for glial thickness measurement. ISN segment before the first major branch point (cyan bar) is used for quantification. Glial cells are labeled by *repo-Gal4>UAS-CD4-tdTomato* and first segmented to generate a binary mask. The thickness of ISN is measured using Local Thickness function in ImageJ.
(TIF)

S3 Fig LOF phenotypes of *Syx5* and SNAP genes at the NMJ (related to Fig 3). (A–B) NMJs of the control (A) and *Snap24/Snap25/Snap29* KO induced by *wor-Cas9* (B), imaged using a lower detection setting. Neurons are shown by HRP staining. Scale bar: 10μm. Related to Fig 3A and 3B. Yellow arrowheads indicate dense puncta inside the presynaptic compartment. (C–D) Boutons of *OK371-Gal4* (C) and *Syx5* KD in neurons by *OK371-Gal4* (D). Scale bar: 10μm. (E) Bouton numbers of *OK371-Cas9* (Fig 5D), *Syx5* KO by *OK371-Cas9* (Fig 5E), *OK371-Gal4* and *Syx5* KD by *OK371-Gal4* (D). ***p≤0.001; *p≤0.05; One-way ANOVA. Each circle represents an NMJ: *Cas9*^*OK371*^, n = 23; *Cas9*^*OK371*^>*Syx5*^*gRNA*^, n = 31; ★*Cas9*^*OK371*^>*Syx5*^*gRNA*^, n = 15; *Gal4*^*OK371*^, n = 32; *Gal4*^*OK371*^>*Syx5*^*RNAi*^, n = 34; *p* values are from multiple comparison test using Bonferroni adjustment. All boutons were from NMJ4 in segments A2-A4. The group with red star contains only NMJs with observable bouton defects. The datasets of *Cas9*^*OK371*^, *Cas9*^*OK371*^>*Syx5*^*gRNA*^, and ★*Cas9*^*OK371*^>*Syx5*^*gRNA*^ are the same as in Fig 3. (F) Penetrance of observable bouton morphology defects in 4 genotypes shown in (E). Numbers indicate the sample size of each genotype. The datasets of *Cas9*^*OK371*^ and *Cas9*^*OK371*^>*Syx5*^*gRNA*^ are the same as in Fig 3.
(TIF)

S4 Fig The KO phenotypes of ESCRT components at the NMJ (related to Fig 4). (A) Nrg distribution at the NMJ of the control (A) and *shrb* KO by *wor-Cas9* (B). Axon membranes are visualized by HRP staining, and Nrg protein is detected by antibody staining. Scale bar: 10μm. Yellow arrows indicate IAV colocalization with Nrg aggregation. (B) neuronal-specific *shrb* KD and *TSG101* KD induced by RNAi. “High” and “Low” panels show the zoomed-in view of the area enclosed by the green box imaged at high and low intensity settings. Blue arrowheads indicate EVs. Pink arrowheads indicate satellite boutons, and green arrows indicate filamentous protrusions formed by presynaptic membrane. Yellow arrows indicate IAVs. Scale bar: 10μm. (C–E) comparison of satellite bouton numbers (C), EV numbers normalized by the presynaptic area (D) and IAV areas normalized by the presynaptic area (E) in ESCRT gene KO versus ESCRT gene KD motoneurons. ***p≤0.001, One-way ANOVA. Each circle represents an NMJ: *OK371-Cas9*, n = 36; *shrb*^*OK371-Cas9*^, n = 41; *TSG101*^*OK371-Cas9*^, n = 23; *OK371-Gal4*, n = 32; *shrb-RNAi*^*OK371-Gal4*^, n = 16; *TSG101-RNAi*^*OK371-Gal4*^, n = 30; between-group *p* values are from multiple comparison test using Bonferroni adjustment. The datasets of *OK371-Cas9*, *shrb*^*OK371-Cas9*^, and *TSG101*^*OK371-Cas9*^ are the same as in Fig 4. (F–G) NMJ morphology in the control (F) and *ALiX* KO (G) motoneurons. Neuronal membrane and EVs are visualized by HRP staining. Inset: zoomed-in view of the area enclosed by the green box. Blue arrowheads indicate the EVs surrounding the presynaptic compartment. Scale bar: 10μm. (H) EV numbers normalized by the presynaptic area in control and *ALiX* KO neurons. t-test, *p* = 0.805. *wor-Cas9*, n = 23; *ALiX*^*wor-Cas9*^, n = 20.
(TIF)

S5 Fig The impacts of *gbb* KD on EV numbers, IAV areas and axial boutons (related to Fig 5). (A) normalized EV number, normalized IAV area, and total bouton numbers in genotypes represented by Fig 5A–5F. ***p≤0.001, **p≤0.01, *p≤0.05, One-way ANOVA. Each circle represents an NMJ: *wor-Cas9*, n = 17; *gbb-RNAi*^*Act-Gal4*^, n = 25; *TSG101*^*wor-Cas9*^, n = 21; *TSG101*^*wor-Cas9*^ */ gbb-RNAi*^*Act-Gal4*^, n = 20; *shrb*^*wor-Cas9*^, n = 36; *shrb*^*wor-Cas9*^ with EV loss, n = 19; *shrb*^*wor-Cas9*^ */ gbb-RNAi*^*Act-Gal4*^ with EV loss, n = 20; between-group *p* values are from multiple comparison test using Bonferroni adjustment. The datasets of *wor-Cas9>gRNA-shrb* and *wor-Cas9>gRNA-TSG101* are the same as in Fig 4. (B) NMJ4 with ESCRT gene KO and *gbb* KD in neuron or muscle. Scale bar: 10 μm. (C) NMJ4 satellite bouton number and total bouton numbers of the genotypes in (B). ***p≤0.001, *p≤0.05, One-way ANOVA. Each circle represents an NMJ: *shrb*^*wor-Cas9*^, n = 36; *shrb*^*wor-Cas9*^ *Gal4*^*OK371*^*>gbb*^*RNAi*^, n = 22; *shrb*^*wor-Cas9*^ *Gal4*^*mef2*^*>gbb*^*RNAi*^, n = 29; *shrb*^*wor-Cas9*^ *Gal4*^*Act5C*^*>gbb*^*RNAi*^ with EV loss, n = 20; *TSG101*^*wor-Cas9*^, n = 22; *TSG101*^*wor-Cas9*^ *Gal4*^*OK371*^*>gbb*^*RNAi*^, n = 30; *TSG101*^*wor-Cas9*^ *Gal4*^*mef2*^*>gbb*^*RNAi*^, n = 36; *TSG101*^*wor-Cas9*^ *Gal4*^*Act5C*^*>gbb*^*RNAi*^ with EV loss, n = 20; between-group *p* values are from multiple comparison test using Bonferroni adjustment. The datasets of *wor-Cas9>gRNA-shrb* and *wor-Cas9>gRNA-TSG101* are the same as in Fig 4; the datasets of *shrb*^*wor-Cas9*^ *Gal4*^*Act5C*^*>gbb*^*RNAi*^ and *TSG101*^*wor-Cas9*^ *Gal4*^*Act5C*^*>gbb*^*RNAi*^ are the same as in Fig 5. (D) NMJ4 of control (left panel) and Tkv-EGFP overexpressing (right panel) neurons. Scale bar: 10 μm. Numbers of satellite boutons in each genotype are quantified. t-test, *p* = 0.614. *OK371-Gal4*, n = 32; *Gal4*^*OK371*^*>Tkv-EGFP*, n = 36. (E) EVs surrounding Tkv-EGFP overexpressing NMJ4 neuron. Postsynaptic Tkv-EGFP colocalizes with postsynaptic HRP signal. Scale bar: 2 μm. (F) Nuclear pMad staining in the ventral nerve cord of the control (left panel), *shrb* KO (middle panel) and *TSG101* KO (right panel). (G) Nuclear pMad levels from experiments in (F). ***p≤0.001, One-way ANOVA. Each circle represents a nucleus: *wor-Cas9*, n = 317; *Cas9*^*wor*^*>shrb*^*gRNA*^, n = 647; *Cas9*^*wor*^*>TSG101*^*gRNA*^, n = 480, between-group *p* values are from multiple comparison test using Bonferroni adjustment. (H) pMad staining in control (upper panel), *shrb* KO (middle panel) and *TSG101* KO (lower panel) at NMJ4 boutons. Scale bar: 5 μm. (I) quantification of synaptic pMad levels from experiments in (H). ***p≤0.001, One-way ANOVA. Each circle represents an NMJ: *wor-Cas9*, n = 18; *Cas9*^*wor*^*>shrb*^*gRNA*^, n = 15; *Cas9*^*wor*^*>TSG101*^*gRNA*^, n = 25, between-group *p* values are from multiple comparison test using Bonferroni adjustment.
(TIF)

S1 Table Key resource table. (XLSX)

S2 Table Sample sizes of negative tester (NT)-Cas9 efficiency test. (DOCX)

S3 Table Numeric data for plots. (XLSX)
