# Supplementary figures and images for: Tissue-specific knockout in the Drosophila neuromuscular system reveals ESCRT’s role in formation of synapse-derived extracellular vesicles
Source: PLoS Genet. 2024 Oct 10;20(10):e1011438. doi: 10.1371/journal.pgen.1011438 (PMC11495600; doi:10.1371/journal.pgen.1011438)

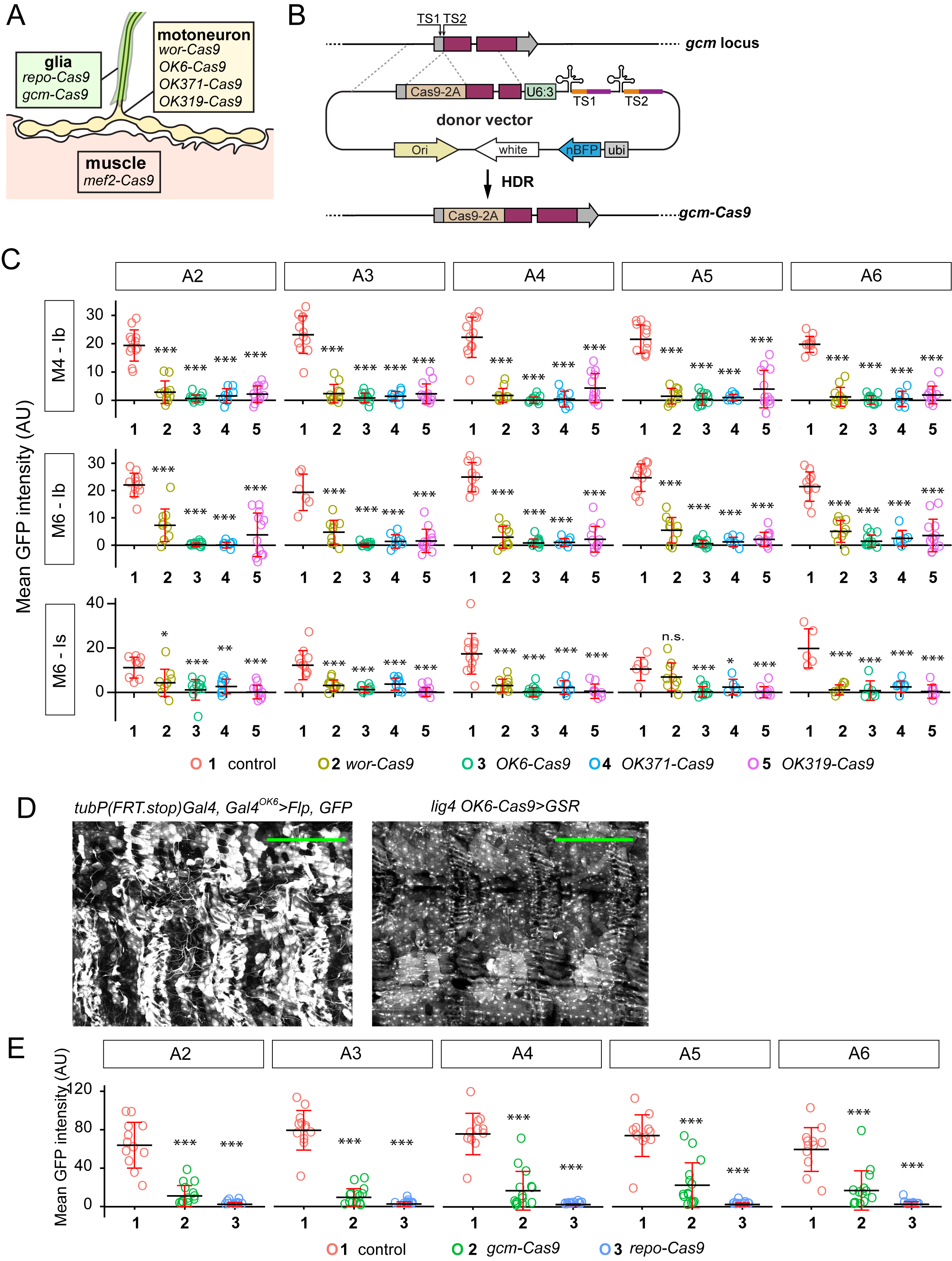

Supplement: S1 Fig — (A) A diagram of the Cas9 lines made for this study and their targeting tissues. (B) A diagram of the generation of gcm-Cas9 by CRISPR-mediated knock-in. A Cas9-2A coding sequence is inserted in-frame immediately after the start codon of gcm. TS1, target site 1. TS2, target site 2. HDR, homology-directed repair. (C) Scatter plots of all data shown in Fig 1A–1C. ***p≤0.001; one-way ANOVA. p values were adjusted by Bonferroni post hoc method. See S2 Table for sample sizes. (D) Comparison of OK6-Gal4 and OK6-Cas9 activity patterns in epidermal cells and trachea on the larval body wall. Activity pattern of OK6-Cas9 is visualized by crossing to lig4; GSR. The non-homologous end joining (NHEJ)-deficient lig4 mutation was combined with GSR to increase the frequency of SSA and thus the reliability of GSR labeling. OK6-Gal4 activity pattern is visualized by tubP(FRT.stop)Gal4, UAS-Flp, GFP. Scale bar: 500 μm. (E) Scatter plots of all data shown in Fig 1D–1E. ***p≤0.001; one-way ANOVA. p values were adjusted by Bonferroni post hoc method. See S2 Table for sample sizes. (TIF) [file pgen.1011438.s001.tif]

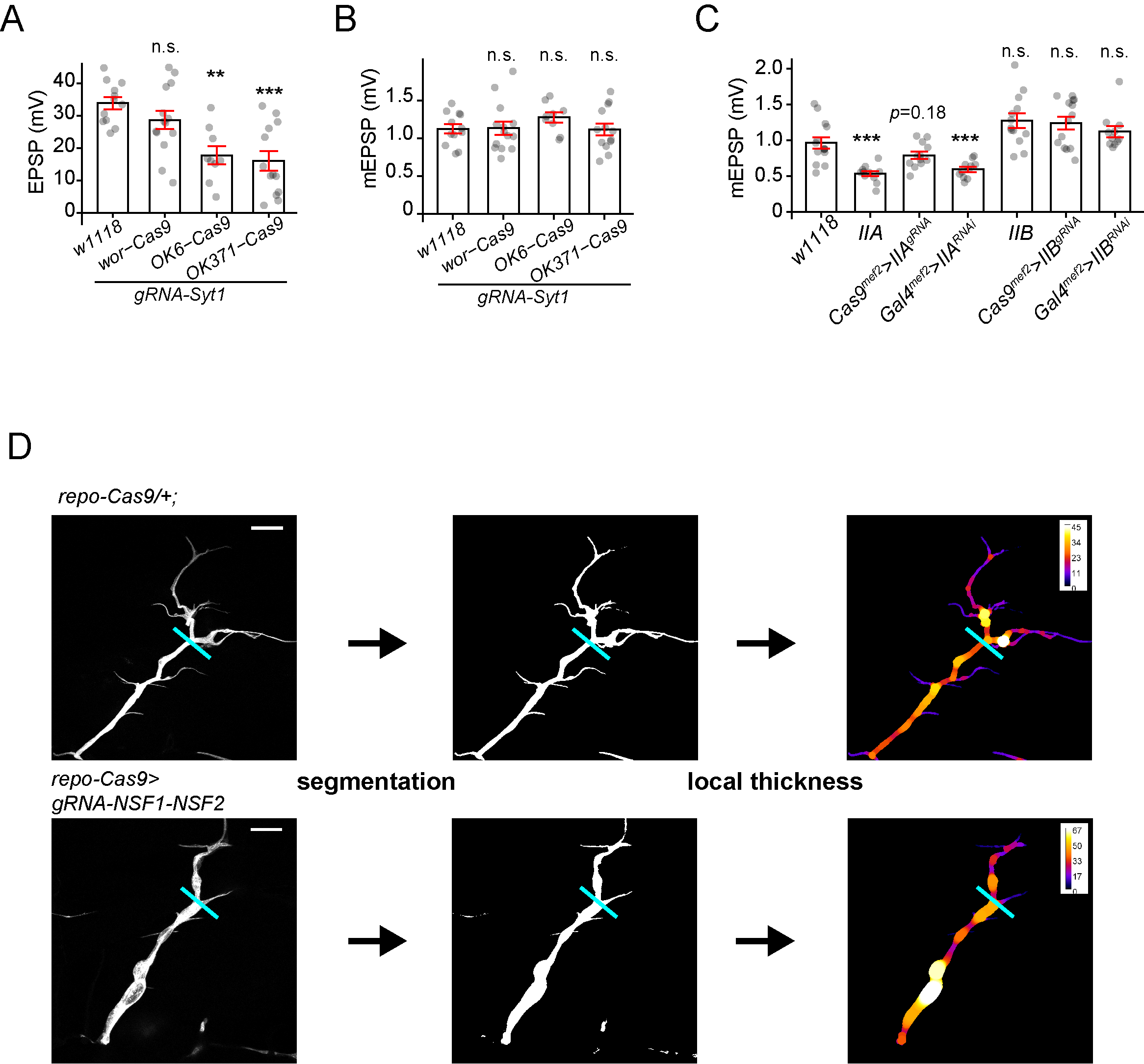

Supplement: S2 Fig — (A) Average evoked EPSP amplitudes comparing the motor neuron Cas9s wor-Cas9/+;gRNA-Syt1, OK6-Cas9/+;gRNA-Syt1, and OK371-Cas9/+;gRNA-Syt1 to w1118 controls. ***p≤0.001; **p≤0.01; One-way ANOVA. Each dot represents an NMJ: w1118, n = 12; wor-Cas9>gRNA-Syt1, n = 14; OK6-Cas9>gRNA-Syt1, n = 9; OK371-Cas9>gRNA-Syt1, n = 9; OK319-Cas9>gRNA-Syt1, n = 13. (B) Average mEPSPs comparing lines listed in (A). No significant difference is found between the lines. One-way ANOVA. Sample sizes are the same as in (A). (C) Comparison of the muscle specific Mef2-Cas9 to other methods of glutamate receptor loss-of-function in affecting mEPSPs. Electrophysiological recordings were conducted on muscles 6 and 7 in segments A2 and A3. Mef2-Cas9/+;gRNA-GluRIIA/+ reduces mEPSP amplitude, as expected, though not significantly nor as robustly as GluRIIApv3 null mutants or Mef2-Gal4/+;UAS-GluRIIA-RNAi. Similarly, Mef2-Cas9/+;gRNA-GluRIIB/+ increases mEPSP amplitude to similar levels as GluRIIBsp5 null mutants and Mef2-Gal4/+;UAS-GluRIIB-RNAi, though again not statistically significantly different from control mEPSP amplitudes of w1118. ***p≤0.001, One-way ANOVA. Each dot represents an NMJ: w1118, n = 12; GluRIIA, n = 11; Mef2-Cas9>gRNA-GluRIIA, n = 12; Mef2-Gal4>GluRIIA-RNAi, n = 11; GluRIIB, n = 13; Mef2-Cas9>gRNA-GluRIIB, n = 14; Mef2-Gal4>GluRIIB-RNAi, n = 11. (D) The method for glial thickness measurement. ISN segment before the first major branch point (cyan bar) is used for quantification. Glial cells are labeled by repo-Gal4>UAS-CD4-tdTomato and first segmented to generate a binary mask. The thickness of ISN is measured using Local Thickness function in ImageJ. (TIF) [file pgen.1011438.s002.tif]

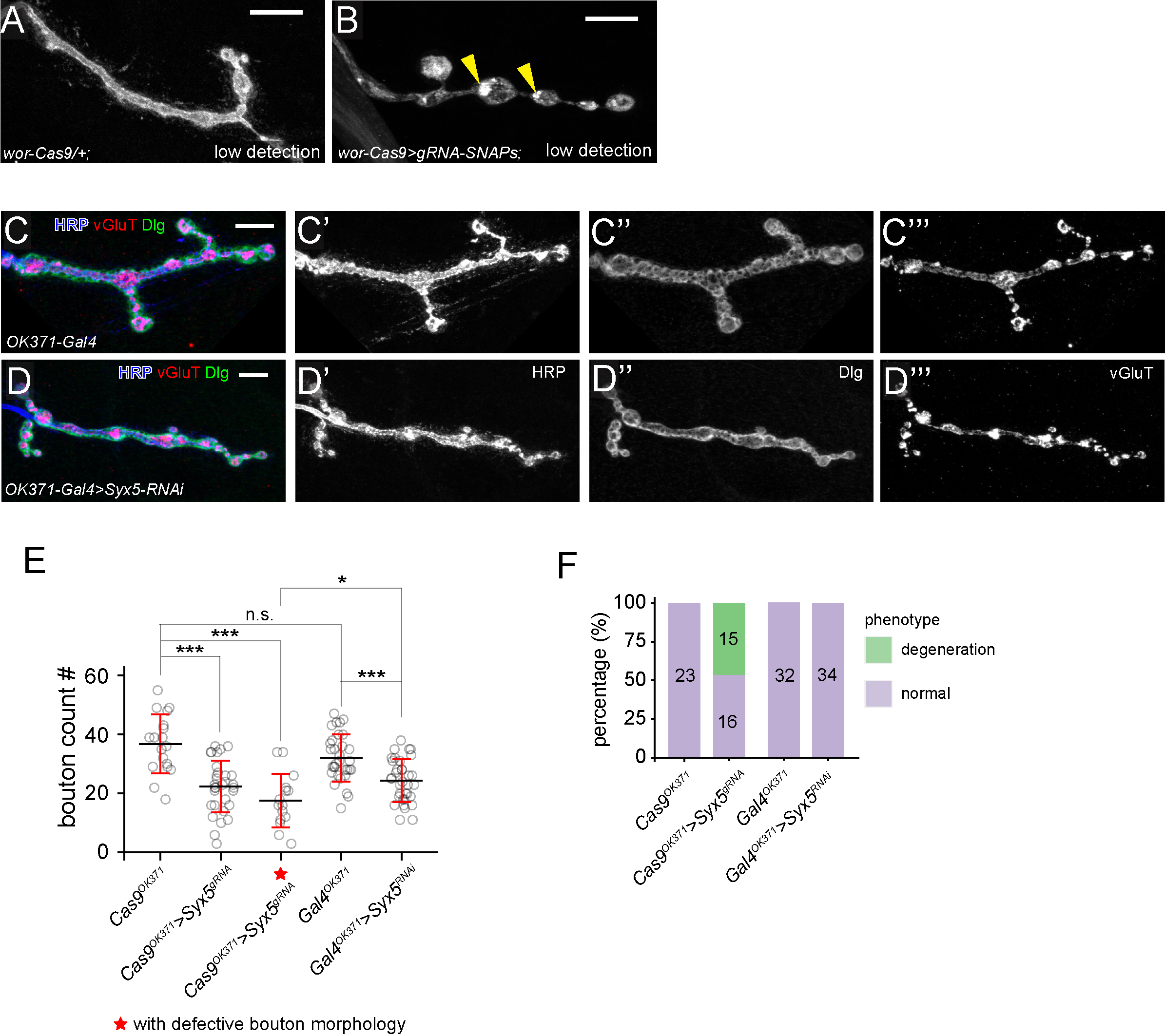

Supplement: S3 Fig — (A–B) NMJs of the control (A) and Snap24/Snap25/Snap29 KO induced by wor-Cas9 (B), imaged using a lower detection setting. Neurons are shown by HRP staining. Scale bar: 10μm. Related to Fig 3A and 3B. Yellow arrowheads indicate dense puncta inside the presynaptic compartment. (C–D) Boutons of OK371-Gal4 (C) and Syx5 KD in neurons by OK371-Gal4 (D). Scale bar: 10μm. (E) Bouton numbers of OK371-Cas9 (Fig 5D), Syx5 KO by OK371-Cas9 (Fig 5E), OK371-Gal4 and Syx5 KD by OK371-Gal4 (D). ***p≤0.001; *p≤0.05; One-way ANOVA. Each circle represents an NMJ: Cas9OK371, n = 23; Cas9OK371>Syx5gRNA, n = 31; ★Cas9OK371>Syx5gRNA, n = 15; Gal4OK371, n = 32; Gal4OK371>Syx5RNAi, n = 34; p values are from multiple comparison test using Bonferroni adjustment. All boutons were from NMJ4 in segments A2-A4. The group with red star contains only NMJs with observable bouton defects. The datasets of Cas9OK371, Cas9OK371>Syx5gRNA, and ★Cas9OK371>Syx5gRNA are the same as in Fig 3. (F) Penetrance of observable bouton morphology defects in 4 genotypes shown in (E). Numbers indicate the sample size of each genotype. The datasets of Cas9OK371 and Cas9OK371>Syx5gRNA are the same as in Fig 3. (TIF) [file pgen.1011438.s003.tif]

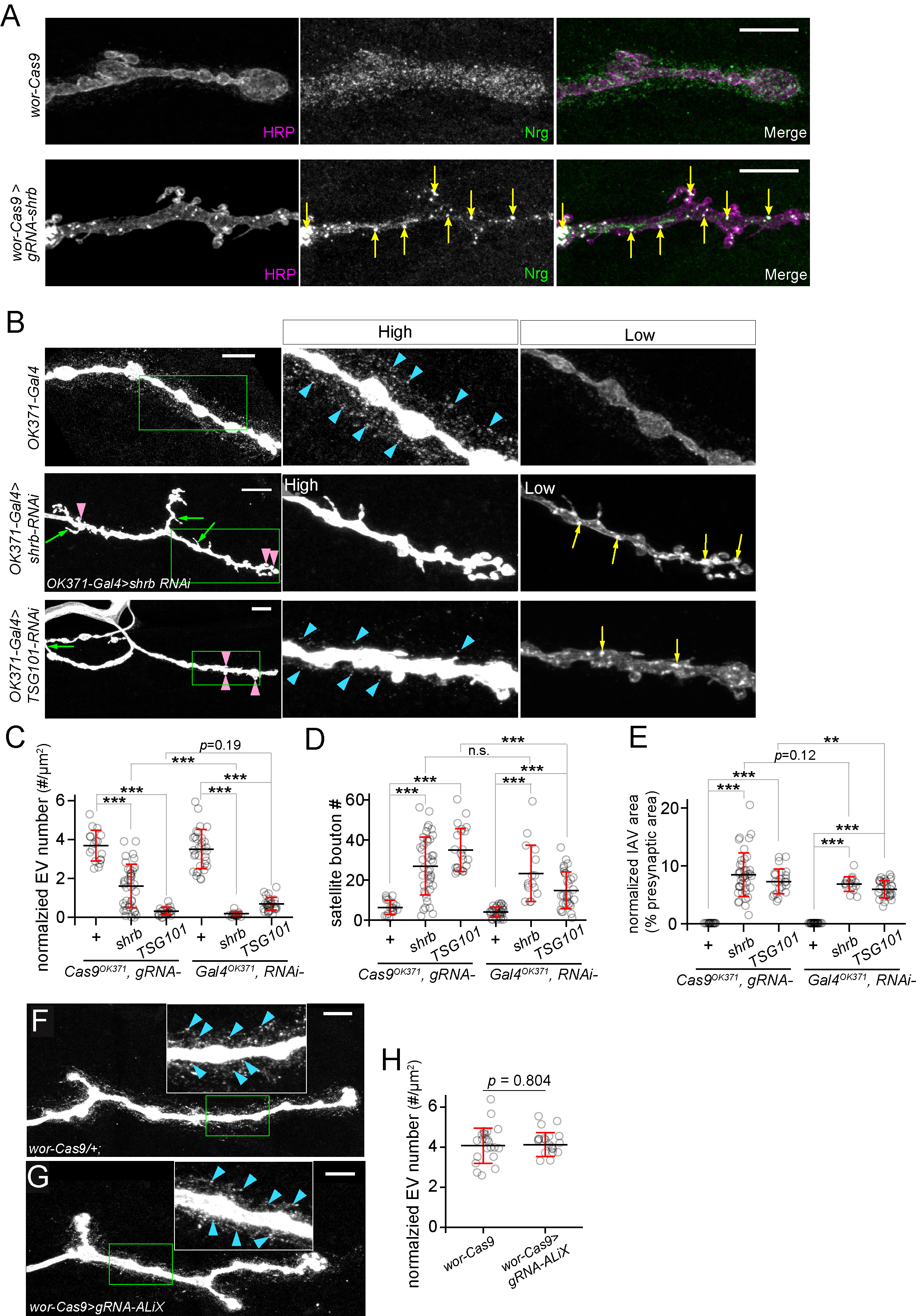

Supplement: S4 Fig — (A) Nrg distribution at the NMJ of the control (A) and shrb KO by wor-Cas9 (B). Axon membranes are visualized by HRP staining, and Nrg protein is detected by antibody staining. Scale bar: 10μm. Yellow arrows indicate IAV colocalization with Nrg aggregation. (B) neuronal-specific shrb KD and TSG101 KD induced by RNAi. “High” and “Low” panels show the zoomed-in view of the area enclosed by the green box imaged at high and low intensity settings. Blue arrowheads indicate EVs. Pink arrowheads indicate satellite boutons, and green arrows indicate filamentous protrusions formed by presynaptic membrane. Yellow arrows indicate IAVs. Scale bar: 10μm. (C–E) comparison of satellite bouton numbers (C), EV numbers normalized by the presynaptic area (D) and IAV areas normalized by the presynaptic area (E) in ESCRT gene KO versus ESCRT gene KD motoneurons. ***p≤0.001, One-way ANOVA. Each circle represents an NMJ: OK371-Cas9, n = 36; shrbOK371-Cas9, n = 41; TSG101OK371-Cas9, n = 23; OK371-Gal4, n = 32; shrb-RNAiOK371-Gal4, n = 16; TSG101-RNAiOK371-Gal4, n = 30; between-group p values are from multiple comparison test using Bonferroni adjustment. The datasets of OK371-Cas9, shrbOK371-Cas9, and TSG101OK371-Cas9 are the same as in Fig 4. (F–G) NMJ morphology in the control (F) and ALiX KO (G) motoneurons. Neuronal membrane and EVs are visualized by HRP staining. Inset: zoomed-in view of the area enclosed by the green box. Blue arrowheads indicate the EVs surrounding the presynaptic compartment. Scale bar: 10μm. (H) EV numbers normalized by the presynaptic area in control and ALiX KO neurons. t-test, p = 0.805. wor-Cas9, n = 23; ALiXwor-Cas9, n = 20. (TIF) [file pgen.1011438.s004.tif]

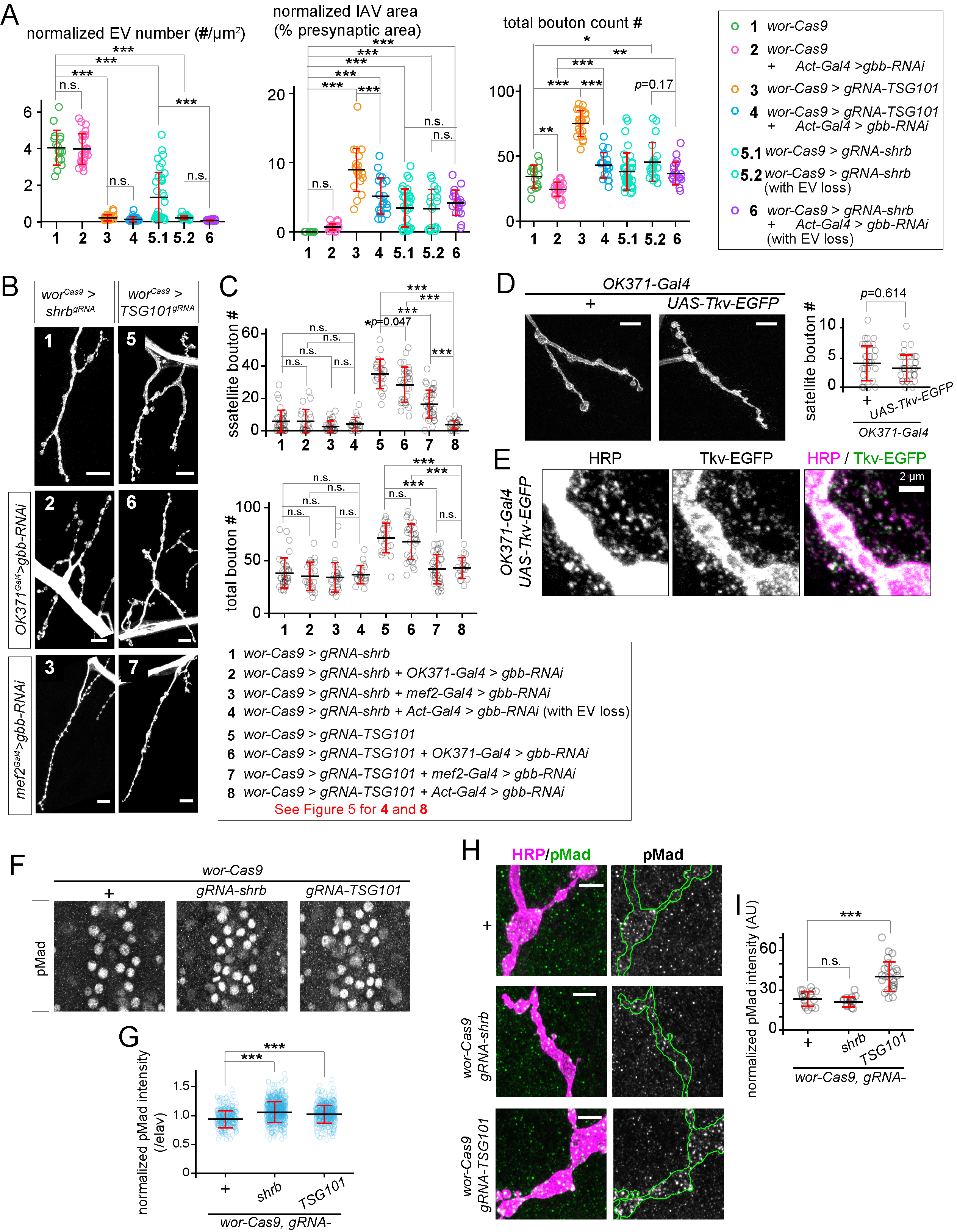

Supplement: S5 Fig — (A) normalized EV number, normalized IAV area, and total bouton numbers in genotypes represented by Fig 5A–5F. ***p≤0.001, **p≤0.01, *p≤0.05, One-way ANOVA. Each circle represents an NMJ: wor-Cas9, n = 17; gbb-RNAiAct-Gal4, n = 25; TSG101wor-Cas9, n = 21; TSG101wor-Cas9 / gbb-RNAiAct-Gal4, n = 20; shrbwor-Cas9, n = 36; shrbwor-Cas9 with EV loss, n = 19; shrbwor-Cas9 / gbb-RNAiAct-Gal4 with EV loss, n = 20; between-group p values are from multiple comparison test using Bonferroni adjustment. The datasets of wor-Cas9>gRNA-shrb and wor-Cas9>gRNA-TSG101 are the same as in Fig 4. (B) NMJ4 with ESCRT gene KO and gbb KD in neuron or muscle. Scale bar: 10 μm. (C) NMJ4 satellite bouton number and total bouton numbers of the genotypes in (B). ***p≤0.001, *p≤0.05, One-way ANOVA. Each circle represents an NMJ: shrbwor-Cas9, n = 36; shrbwor-Cas9 Gal4OK371>gbbRNAi, n = 22; shrbwor-Cas9 Gal4mef2>gbbRNAi, n = 29; shrbwor-Cas9 Gal4Act5C>gbbRNAi with EV loss, n = 20; TSG101wor-Cas9, n = 22; TSG101wor-Cas9 Gal4OK371>gbbRNAi, n = 30; TSG101wor-Cas9 Gal4mef2>gbbRNAi, n = 36; TSG101wor-Cas9 Gal4Act5C>gbbRNAi with EV loss, n = 20; between-group p values are from multiple comparison test using Bonferroni adjustment. The datasets of wor-Cas9>gRNA-shrb and wor-Cas9>gRNA-TSG101 are the same as in Fig 4; the datasets of shrbwor-Cas9 Gal4Act5C>gbbRNAi and TSG101wor-Cas9 Gal4Act5C>gbbRNAi are the same as in Fig 5. (D) NMJ4 of control (left panel) and Tkv-EGFP overexpressing (right panel) neurons. Scale bar: 10 μm. Numbers of satellite boutons in each genotype are quantified. t-test, p = 0.614. OK371-Gal4, n = 32; Gal4OK371>Tkv-EGFP, n = 36. (E) EVs surrounding Tkv-EGFP overexpressing NMJ4 neuron. Postsynaptic Tkv-EGFP colocalizes with postsynaptic HRP signal. Scale bar: 2 μm. (F) Nuclear pMad staining in the ventral nerve cord of the control (left panel), shrb KO (middle panel) and TSG101 KO (right panel). (G) Nuclear pMad levels from experiments in (F). ***p≤0.001, One-way ANOVA. Each circle re [file pgen.1011438.s005.tif]
