# Supplementary material for: Tissue-specific knockout in the Drosophila neuromuscular system reveals ESCRT’s role in formation of synapse-derived extracellular vesicles
Source: PLoS Genet. 2024 Oct 10;20(10):e1011438. doi: 10.1371/journal.pgen.1011438 (PMC11495600; doi:10.1371/journal.pgen.1011438)
Supplement: S2 Table — (DOCX) [file pgen.1011438.s007.docx]

**Table S2. Sample sizes of negative tester (NT)-Cas9 efficiency test**

| **group** | **A2** | **A3** | **A4** | **A5** | **A6** |
| --- | --- | --- | --- | --- | --- |
| NMJ4 NT | 12 | 12 | 12 | 12 | 10 |
| NMJ4 *wor-Cas9* | 10 | 10 | 10 | 9 | 10 |
| NMJ4 *OK6-Cas9* | 12 | 12 | 12 | 12 | 12 |
| NMJ4 *OK371-Cas9* | 8 | 8 | 8 | 7 | 7 |
| NMJ4 *OK319-Cas9* | 12 | 12 | 12 | 12 | 12 |
| NMJ6/7 Ib NT | 12 | 11 | 12 | 12 | 12 |
| NMJ6/7 Ib *wor-Cas9* | 10 | 10 | 10 | 10 | 10 |
| NMJ6/7 Ib *OK6-Cas9* | 12 | 11 | 12 | 12 | 12 |
| NMJ6/7 Ib *OK371-Cas9* | 8 | 8 | 7 | 7 | 7 |
| NMJ6/7 Ib *OK319-Cas9* | 12 | 12 | 12 | 11 | 12 |
| NMJ6/7 Is NT | 9 | 11 | 12 | 6 | 5 |
| NMJ6/7 Is *wor-Cas9* | 9 | 10 | 9 | 9 | 7 |
| NMJ6/7 Is *OK6-Cas9* | 11 | 9 | 12 | 11 | 6 |
| NMJ6/7 Is *OK371-Cas9* | 8 | 8 | 7 | 5 | 7 |
| NMJ6/7 Ib *OK319-Cas9* | 12 | 12 | 8 | 9 | 8 |
| Glia NT | 12 | 12 | 11 | 12 | 12 |
| Glia *gcm-Cas9* | 14 | 14 | 14 | 14 | 14 |
| Glia *repo-Cas9* | 12 | 12 | 12 | 11 | 11 |
